# Gut microbiota shaping fatty liver disease progression

**DOI:** 10.26508/lsa.202603756

**Published:** 2026-10-12

**Authors:** Martyna Wysokińska, Marta Mysza, Aleksandra E Uryga, Aleksandra A Kołodziejczyk

**Affiliations:** Laboratory of Cellular Genomics, International Institute of Molecular and Cell Biology , Warsaw, Poland

## Abstract

The gut–liver axis is an important regulator of homeostasis, and its dysregulation has been implicated in the pathogenesis of metabolic dysfunction–associated steatotic liver disease (MASLD). In this review, we summarize current knowledge on both compositional alterations of the gut microbiome in MASLD and the functional consequences of these changes. We first discuss changes across major microbial domains, including bacteria, archaea, eukaryota, and bacteriophages, and then focus on microbiota-derived metabolites, such as short-chain fatty acids, trimethylamine and its derivative trimethylamine N-oxide, tryptophan-derived indoles, secondary bile acids, and other bioactive compounds. Their complex impacts on intestinal barrier integrity, inflammation, lipid and glucose metabolism, and immune and hormonal signaling in the liver are highlighted. We further examine mechanisms involving the translocation of microbial products and the modulation of nuclear and membrane receptors. Finally, we outline current challenges and knowledge gaps that need to be addressed to better understand the role of the gut microbiota in MASLD.

## Abstract (precis)

Gut microbiota and its metabolites shape MASLD progression by modulating intestinal barrier integrity, inflammation, metabolism, and liver signaling.

## Graphical Abstract

**Figure:**
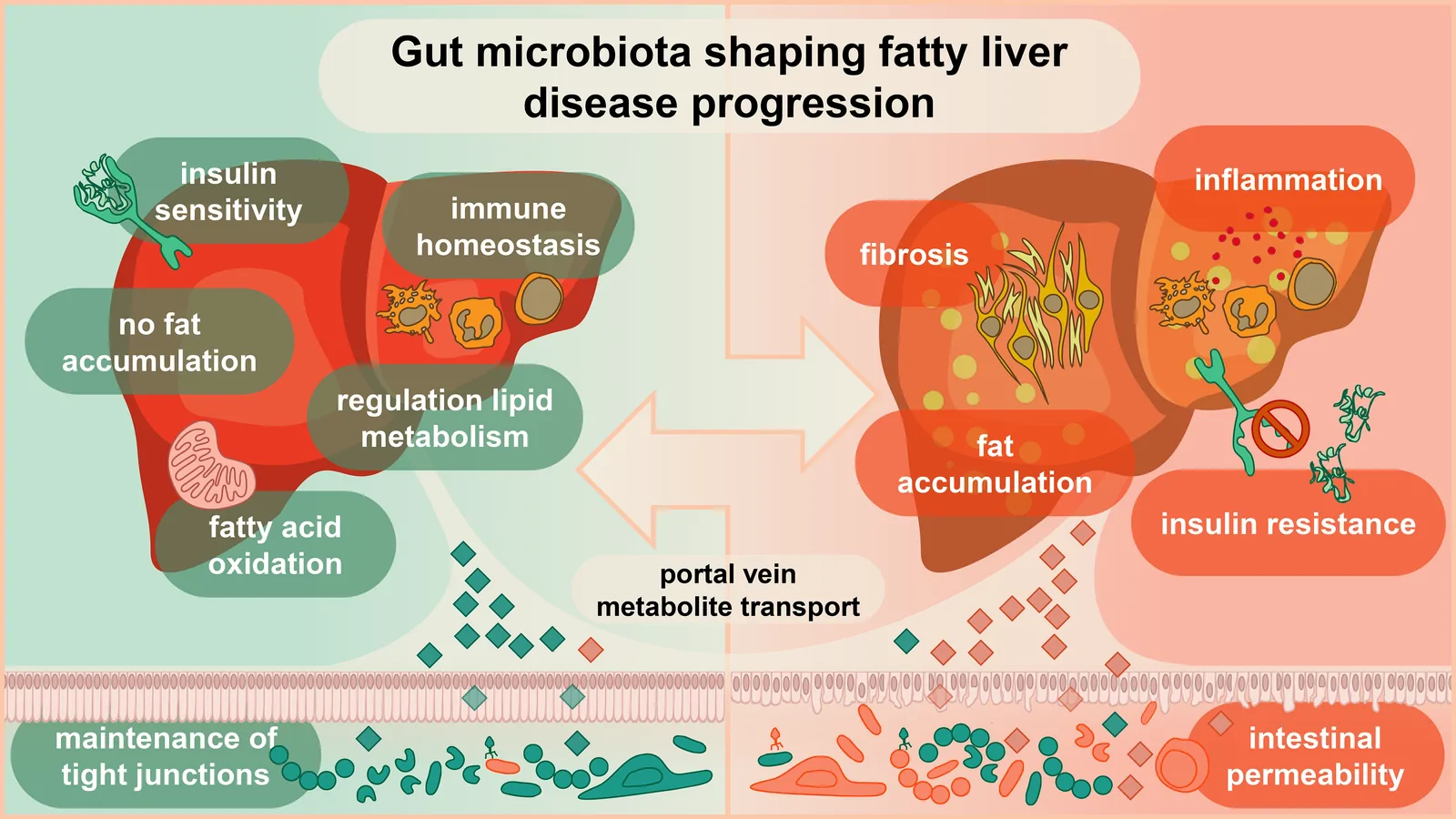

## Introduction

Metabolic dysfunction–associated steatotic liver disease (MASLD) is the most common chronic liver disease worldwide and contributes to a growing health burden (1). MASLD encompasses a spectrum of liver abnormalities marked by excessive fat accumulation in the liver in individuals with no etiological factors other than the presence of features of metabolic syndrome (2). Current estimates suggest that MASLD affects ∼30–40% of the world’s population, and its prevalence is steadily increasing, in parallel with rising rates of obesity, type 2 diabetes, and metabolic syndrome (1). As fatty liver disease is usually asymptomatic, it often remains underdiagnosed and untreated, leading to disease progression. Over time, this condition can develop into metabolic dysfunction–associated steatohepatitis (MASH), cirrhosis, and hepatocellular carcinoma, constituting a major cause of liver-related morbidity and mortality (3, 4, 5).

Previously, the MASLD condition was referred to as nonalcoholic fatty liver disease (NAFLD), and its progressive inflammatory stage as nonalcoholic steatohepatitis (NASH). However, the recent Delphi expert consensus meeting changed the terminology to more accurately reflect the underlying metabolic factors of the disease (2). The term MASLD was introduced to emphasize the key role of metabolic dysfunction in the pathogenesis of the disease, whereas MASH replaces the previously used term NASH. It is important to bear in mind that although about 99% of patients previously diagnosed with NAFLD are reclassified as MASLD, some cases are not. These include drug-induced fatty liver and steatosis that accompanies conditions (6). Given the near-complete overlap between MASLD and NAFLD, this study uses the new terminology throughout, even where the original publications used different terms.

The pathogenesis of MASLD is complex and multifactorial, involving the interplay of genetic, metabolic, and environmental factors. Key mechanisms involved in disease development include insulin resistance, lipid metabolism disorders, oxidative stress, mitochondrial dysfunction, and chronic low-grade inflammation (3). These processes contribute to hepatic lipid accumulation and subsequent liver cell damage, ultimately driving disease progression (7). Despite extensive research in this field, the new mechanisms determining the initiation, progression, and interindividual variability of the disease are still being discovered. Among environmental factors, diet plays a particularly important role in the development and progression of MASLD (8). Dietary patterns characterized by high intake of saturated fats, refined carbohydrates, and foods high in fructose syrups are strongly associated with fatty liver disease and metabolic disorders (9, 10). In turn, dietary components such as dietary fiber, polyphenols, and unsaturated fatty acids may exert a protective effect (11, 12). Importantly, diet not only directly influences hepatic metabolism but also significantly shapes the composition and metabolic activity of the gut microbiota (13, 14), suggesting an additional, indirect pathway through which dietary factors may contribute to liver disease.

Consequently, increasing attention is being paid to the gut–liver axis. This bidirectional communication system connects the gastrointestinal tract with the liver via anatomical, metabolic, and immunological pathways (15). Through portal circulation, nutrients and microbial products reach the liver directly (16). The human gastrointestinal tract harbors a highly complex and dynamic microbial ecosystem composed of trillions of microorganisms, including bacteria, archaea, bacteriophages, and fungi. Collectively referred to as the gut microbiota, these microbial communities play a crucial role in host physiology by participating in food digestion, modulation of immune responses, maintenance of intestinal barrier function, and regulation of systemic metabolic processes (17, 18). Over the past decade, the number of studies examining the relationship between gut microbiota and liver disease has increased significantly. These studies have provided two complementary perspectives: the characterization of compositional alterations in the gut microbiota associated with MASLD and the investigation of functional consequences of these alterations, particularly through microbially derived metabolites and their effects on the gut–liver axis (19, 20).

Differences in study design, sequencing methodologies, dietary patterns, geographic populations, drug use, and metabolic characteristics of the organism studied may contribute to the variability and inconsistency in the reported results. Furthermore, the complex interactions between diet, metabolism, microbial communities, and microbial metabolites pose a significant challenge in establishing causal relationships. Therefore, the aim of this review is to summarize the current evidence regarding the role of gut microbiota and bacterial metabolites in MASLD. Where relevant, we also draw on evidence from other liver diseases with mechanisms likely to overlap with MASLD. Moreover, this review identifies current limitations and future directions for research in this rapidly evolving field.

## Bacteria Associated With MASLD

MASLD and MASH are quite consistently associated with a loss of microbial alpha-diversity (21, 22). Several human studies aimed at identifying bacterial species associated with MASLD, MASH, or features of these diseases, such as the extent of fibrosis, often show different microbiota patterns. These differences are likely the result of study design, cohort composition, and geographical differences in microbiota. In the following sections, we provide a narrative overview of selected bacterial taxa that have been most extensively investigated in relation to MASLD.

### *Bacteroides* spp.

*Bacteroides,* one of the most abundant genera in the human microbiome, was shown to be significantly associated with MASH, regardless of metabolic factors (i.e., metabolic syndrome, body mass index (BMI), diabetes, blood pressure, triglyceride levels, HDL cholesterol levels). Although this study was done on a relatively small cohort of 57 participants, the authors also observed that the abundance of this genus is also correlated with the extent of fibrosis staged by liver biopsy (23).

As *Bacteroides* is a heterogeneous genus encompassing tens of different species that colonize the human gut and may contribute to the disease in different ways, it is relevant to zoom deeper than the genus level. Indeed, in patients with biopsy-proven NAFLD, the abundance of some species such as *Bacteroides vulgatus, Bacteroides ovatus,* and *Bacteroides stercoris* was increased, whereas that of others, such as *Bacteroides dorei*, was decreased (24). The expansion of *B. stercoris* in MASLD was replicated in a randomized clinical trial (25). It showed that *B. stercoris* levels and activity of liver enzymes (alanine aminotransferase (ALT), aspartate aminotransferase (AST)) in blood and triglyceride content in the liver can be lowered using dietary intervention with resistant starch (25). The mouse study in high-fat, high-choline model showed that increase in *B. stercoris* leads to intrahepatic fat deposition, inflammation, and fibrosis. The inspection of the blood and stool contents placed lipopolysaccharide (LPS) and branched-chain amino acids (BCAAs), in particular, valine, as candidates that are exacerbating MASLD progression (24, 25). These results led to attempts to identify potential probiotics that would outcompete *B. stercoris* and therefore improve MASLD outcomes. For example, *Bacteroides sp.* 4_1_36 (B6) strain has shown in vitro antimicrobial activity against *B. stercoris* DSM 19555, as well as promotes fatty acid clearance in hepatocytes (26).

Similarly, in a mouse model of MASLD induced by a high-fat diet (HFD), supplementation with *Bacteroides thetaiotaomicron* (*B. theta*) led to alleviation of steatosis, as well as changes in other parameters associated with metabolic syndrome, including improved insulin sensitivity, and reduced cholesterol and triglyceride levels (27). The decrease in steatosis was also observed in a mouse model of alcoholic liver disease when supplemented with *B. theta* (28). The proposed mechanism driving improvements in *B. theta* supplementation relies not only on an increase in the diversity of microbiota, but also on enhanced intestinal folate biosynthesis, as indicated by increased folate levels in feces and liver (27).

### *Prevotella* spp.

In four independent cohorts, including both adults and children, a high abundance of *Prevotella copri* was associated with moderate and advanced fibrosis in MASH (29, 30, 31, 32). This association was observed regardless of the etiology of chronic liver disease and after considering confounding factors persisted (32). However, these findings should be interpreted with caution, as three of the four cohorts consisted primarily of Hispanic participants (29, 30, 31). Ethnicity and associated dietary habits are known to influence gut microbial composition and may therefore contribute to the observed associations.

Many bacteria, including *P*. *copri*, exhibit substantial strain-level genetic and functional diversity (23, 33, 34, 35). These strain-level differences are rarely studied; hence, the conclusions about species or even higher taxonomic ranks need to be taken with caution. The mechanism through which *P. copri* interacts with the host includes activation of the inflammatory response via TLR2 signaling and promoting the Th17 polarization (32). The abundance of *Prevotella* was also shown to be positively correlated with even moderate alcohol consumption, which may constitute an additional mechanism promoting liver damage in MASLD (29). However, causal relationships were not sufficiently established.

### *Ruminococcus* spp.

The *Ruminococcus* genus is associated with lower levels of steatosis in two large independent cohorts of individuals with MASLD (36, 37, 38). The causative link between *Ruminococcus* and better clinical outcomes was also demonstrated in mouse models. In animals fed a methionine- and choline-deficient diet, colonization by *Ruminococcus faecis* led to decreased ALT and AST activity and regression of fibrosis, accompanied by an increase in the concentration of secondary bile acids (BAs) in the cecum. The authors propose that the mechanism through which *R. faecis* elicits its positive effects is related to the modulation of the secondary BA pool and, through that, influences inflammatory and fibrotic processes in the liver (37).

Another member of the *Ruminococcus* genus, namely, *R. torques*, was associated with decreased activity of liver enzymes in blood, a marker of liver injury in MASLD patients undergoing time-restricted feeding (39). Colonization with *R. torques* in mouse models of diet-dependent fatty liver disease led to reduced steatosis, lower hepatic triglyceride and ceramide level, and decreased ALT and AST activity. Notably, the improvement in lipid and ceramide parameters preceded changes in body weight, providing an argument for a mechanism independent of weight loss. The proposed mechanism involves suppression of the intestinal HIF-2α-ceramide pathway by 2-hydroxy-4-methylpentanoic acid produced by *R. torques* (39).

### Klebsiella pneumoniae

Levels of *Klebsiella pneumoniae* are only sporadically observed to be higher in MASLD or MASH (40). Nevertheless, this bacterium is of particular interest because some of its strains have high alcohol production capacity. These strains, denoted as HiAlc-*Kpn*, metabolize monosaccharides to pyruvate, which is then converted to ethanol via mixed and 2,3-butanediol fermentation pathways (41, 42). Colonization of mice with HiAlc-*Kpn* increased their blood ethanol concentrations from undetectable to approximately half the concentration observed in mice administered by gavage 4 ml of pure ethanol per kilogram of body weight (41). This shows that bacterial ethanol production in some cases is not negligible. Colonization of both specific-pathogen-free and germ-free mice with HiAlc-*Kpn* induced hepatic steatosis, elevated aminotransferase activity, oxidative stress, and inflammation, supporting a potential causal role for these strains in MASLD rather than a simple association (40, 43). The negative impact of ethanol on hepatocytes has been well documented and extensively revised elsewhere (44).

In MASLD and MASH patients, regardless of minor differences in total *K. pneumoniae* levels, their alcohol-producing capacity was significantly higher than in healthy controls, suggesting that extensive alcohol production is specific to subset of strains (40).

Negative effects can be reversed by targeting HiAlc-*Kpn* with lytic phage *phiW14.* In this intervention, the decrease in levels of HiAlc-*Kpn* did not significantly impact the microbiota composition, but decreased steatosis and inflammation in mouse models (45). Interestingly, beyond ethanol production, HiAlc-*Kpn* affects the gut–liver axis also through modulation of intestinal permeability, which was shown in mice by increased intestinal permeability markers, including DAO and serum D-lactate, and reduced expression of tight junction proteins such as occludin and ZO-1 (40, 41). It is important to note that the strain-level differences are crucial, and changes in bacterial abundance alone are not sufficient to induce the disease phenotype (46).

Although in the context of liver disease HiAlc-*Kpn* is the main ethanol-producing culprit, studies on auto-brewery syndrome showed an overrepresentation of ethanol fermentation pathway genes also in other human gut bacterial species (46). Moreover, not all studies identify *K. pneumoniae* as an ethanol producer in vivo (47). However, these discrepancies may originate from variable strain characteristics in different populations and suggest that high alcohol production may be a relatively rare trait in *K. pneumoniae.*

### *Escherichia* spp.

Studies conducted in adults and children indicate an increased relative abundance of *Escherichia* in people with MASH (38, 48). In the pediatric cohort, *Escherichia* abundance was not significantly different between healthy and obese children but was elevated in obese children with NASH in comparison with obese children without it. This resulted in the hypothesis that similar to closely related *K. pneumoniae, Escherichia* produces ethanol, which, upon absorption, damages the liver (49). According to metagenomic analysis, *Escherichia sp.* Y64 was associated with liver injury and dyslipidemia in MASH (43).

Experimental data in mouse models support the pathogenic potential of selected *E. coli* strains. *E. coli* strain NF73-1, isolated from patients with MASH, can disrupt the integrity of the gut vascular barrier by inhibiting Wnt/β-catenin signaling in endothelial cells and increasing bacterial translocation to the liver (50). Another of this genus, *Escherichia fergusonii*, was also demonstrated to induce MASLD in the rat model. It caused triglyceride accumulation, increased inflammation, and early fibrosis without weight gain (51).

### Enterococcus faecium

Similar to *Klebsiella* and *Escherichia,* the impact of *Enterococcus* on the liver is strain-specific. *Enterococcus faecium* B6 was identified as significantly associated with MASLD in obese children (43). Studies in animals demonstrated that, indeed, this bacterium leads to increased steatosis in mouse models. Functional analysis showed that via the *mfnA* gene, *E. faecium* B6 produces a bioactive metabolite, tyramine, which activates the PPAR-γ receptor in hepatocytes. This in turn leads to lipid accumulation, inflammation, and, as a result, liver fibrosis (52, 53).

### Faecalibacterium prausnitzii

*Faecalibacterium prausnitzii*, a dominant member of the healthy adult human gut microbiota, has emerged as a marker of balanced gut microbiota owing to its consistently reduced abundance in patients with inflammatory bowel disease (54). More recently, evidence from studies in patients with MASLD has also supported the notion of *F. prausnitzii* as a potential microbial biomarker of the disease.

At the genus level, *Faecalibacterium* was negatively correlated with MASLD in independent cohorts (55, 56, 57). Similarly, at the strain level *F. prausnitzii* was reduced in MASLD and MASH patients independent of BMI and the presence of insulin resistance (58). In addition, in a large biopsy-confirmed MASH cohort (REGENERATE study), *F. prausnitzii* was the only one among 12 significantly differentially abundant species that associated with a “healthy” phenotype after adjusting for age and BMI (48). Disease progression from MASLD to MASH and cirrhosis is accompanied by a progressive decline in *F. prausnitzii* abundance (59).

In mouse models, it was demonstrated that *F. prausnitzii* has hepatoprotective function (60, 61). The proposed mechanism is related to its short-chain fatty acid (SCFA) production, in particular, butyrate. In the context of the gut–liver axis, anti-inflammatory and colonocyte-trophic effects of butyrate improve gut barrier integrity and play a key role in limiting the translocation of bacterial components, including LPS, into the bloodstream and subsequently the liver, where they can activate inflammatory pathways (62).

### Akkermansia muciniphila

Another bacterial strain that is widely associated with beneficial effects is another SCFA producer, *Akkermansia muciniphila* (63). In mouse models, induction of MASLD through a HFD leads to a decrease in the number of *A. muciniphila* (64, 65, 66). Administration of *A. muciniphila* to mice with MASLD because of the HFD led to a reduction in body weight, a decrease in triglycerides, a reduction in hepatic lipid accumulation, suppression of lipogenesis, and inflammation (64, 66). In parallel, improved intestinal barrier integrity was observed, as evidenced by increased mucus layer thickness, increased ZO-1 expression, and decreased serum LPS concentration (64). The mechanism through which this is achieved is linked to the function of SCFA. Moreover, the *A. muciniphila* outer membrane protein Amuc_1100 activates TLR2 signaling, which contributes to enhanced gut mucosal barrier integrity and regulates immunological homeostasis (67, 68). Positive effects are also mediated through mechanisms beyond those involving the gut barrier. *A. muciniphila* secretes P9 protein that induces glucagon-like peptide-1 (GLP-1) secretion by L cells, which in turn ameliorates metabolic disease in mouse models (69). It was also proposed to increase L-aspartate (L-Asp) transport from the intestine to the liver, leading to enhanced β-oxidation and reduction of oxidative stress (70). Notably, human studies have shown reduced *Akkermansia* abundance in patients with MASLD compared to healthy individuals (71, 72). However, in a clinical trial, 3-mo supplementation with *A. muciniphila* reduced activity levels of liver enzymes, further suggesting a causal link (73). Furthermore, patients with alcoholic steatohepatitis had reduced fecal *A. muciniphila* counts compared with healthy controls, and prophylactic administration of *A. muciniphila* in an experimental model reduced liver damage, steatosis, and neutrophil infiltration (74).

### *Dubosiella* spp.

*Dubosiella* depletion was shown to be associated with MASLD in Western diet mouse model (75) and with progression to MASH (65). It has also been suggested as a contributor of the beneficial effects of exercise, such as reduced hepatic lipid accumulation, lower blood triglycerides, and decreased liver enzyme activity in a model of HFD-fed mice. In this context, *Dubosiella* was proposed to act on liver metabolism through modulation of fibroblast growth factor 21 (76). Moreover, oral administration of *Dubosiella newyorkensis* NYU-BL-A4 reduced steatosis, inhibited lipogenesis, and enhanced β-oxidation in the liver (76).

### *Lactobacillus* spp.

A few studies in adults and adolescents have revealed a correlation between reduced *Lactobacillus* spp. abundance and the presence of MASLD (38, 77). As *Lactobacillus* spp. are currently classified as probiotics, some members of the *Lactobacillus* genus were used in interventional studies in humans. In a small group of 20 participants with cholestatic liver disease, supplementation with *L. acidophilus* for 14 d led to a reduction in liver enzyme activity in blood (78). Similarly, in an alcoholic liver disease cohort, levels of *L. acidophilus* were also lower than in healthy controls, and its supplementation has been shown to confer beneficial effects, including reduced serum LPS concentration, ALT and AST levels, and hepatic steatosis (79).

In a mouse model of liver fibrosis induced by bile duct ligation, the relative abundance of *Lactobacillus*, including *L. acidophilus*, correlated with a milder disease phenotype. Moreover, *L. acidophilus* supplementation led to a reduction in hepatocyte necrosis and inflammatory response (77). Similarly, in a mouse model of MASLD-associated hepatocellular carcinoma, *L. acidophilus* was one of the most depleted species, and supplementation with this bacterium alleviated disease (80).

It was suggested that the mechanism through which these effects are mediated is linked to the modulation of BA metabolism in the gut. Altered deconjugation of primary BAs led to changes in activation of the farnesoid X receptor (FXR)–FGF15–CYP7A1 axis and production of primary BAs in the liver (78). The protective effect of *L. acidophilus* may also result from the production of valeric acid, which strengthens the integrity of the intestinal barrier and, through activation of GPR41/43 receptors, inhibits Rho GTPase signaling in hepatocytes (79).

### Helicobacter hepaticus

*Helicobacter hepaticus* is considered a pathobiont because under specific conditions, it can promote disease including colorectal cancer. It was first isolated from the livers of mice with chronic hepatitis, suggesting that under conditions of increased intestinal permeability, the bacterium can translocate from the gut to the liver (81, 82). In mouse models, where *H*. *hepaticus* was introduced intragastrically via oral gavage, it promoted the early development of microvesicular hepatocyte steatosis. Experimental studies have demonstrated that the *H. hepaticus*-derived CdtB toxin can reach the liver and be internalized by hepatocytes, resulting in mitochondrial dysfunction, impaired β-oxidation, and enhanced de novo lipogenesis (82, 83). In addition, patients with MASLD or MASH colonized by *H. hepaticus* were reported to have higher ALT and AST levels. However, these findings were based on a small cohort of 28 patients and require confirmation in larger studies (82).

### Other bacteria

Bacteria that exacerbate disease are typically linked to proinflammatory potential, disruption of the intestinal barrier, and contribution to endotoxemia, BA metabolism, and production of hepatotoxins, especially ethanol (56). In addition, *Streptococcus*, including *Streptococcus salivarius*, *Streptococcus parasanguinis*, and representatives of *Enterobacteriaceae* and *Megasphaera*, are enriched in MASH patients and have been proposed as microbial markers of disease severity (38, 48, 84). Nevertheless, the available evidence does not yet establish the mechanisms by which these taxa may contribute to disease progression, and causal relationships remain to be confirmed.

Taxa reported as hepatoprotective are typically linked to intestinal barrier integrity, SCFA production, or modulation of BA profile. Consistently, *Faecalibacterium*, *Subdoligranulum*, *Haemophilus*, and *Roseburia* were negatively associated with MASLD in human cohort (55). Reduced abundance in MASLD has also been reported in humans for bacteria such as *Mitsuokella* (36)*, Pseudoruminococcus massiliensis* (85), *Intestinibacter bartlettii, Eggerthella lenta, Clostridium bolteae* (86), *Dialister invisus*, and *Ruminococcus* (36, 85). However, it is unclear whether the reduction in abundance of those taxa contributes to the disease.

Also *Dysosmobacter* *welbionis* is less abundant for humans with MASLD and further reduced in individuals with fibrosis (86). Supplementation with the *D. welbionis* J115T in mouse model led to a significant reduction in liver lipid droplet accumulation, supporting a potential hepatoprotective effect (87). Similarly, supplementation with bacteria established causal relationship for *Lactococcus lactis* (formerly *Lactobacillus lactis*), which led to a significant reduction in ALT and improvement in liver health parameters, without significant changes in gut microbiota diversity in a human double-blind, placebo-controlled study (88).

It is worth mentioning that there are also potentially hepatoprotective bacteria that are specifically depleted in individuals with MASLD and normal BMI. In particular, the relative abundance of *Eubacterium hallii, Eubacterium ramulus*, and *Agathobacter rectalis* (formerly *Eubacterium rectale*) is lower in those patients (89, 90). However, in a 120-participant cohort from Inner Mongolia, *Eubacterium rectale* was higher in individuals with hepatic steatosis, suggesting an underlying complexity (85).

Many other bacterial genera also show variable results, particularly between organisms and between disease settings. The genus *Bifidobacterium*, and in particular *Bifidobacterium bifidum*, was more abundant in humans with MASLD and MASH. However, in a Western diet–fed mouse model, their abundance was reduced (91). Moreover, in an interventional human study, lactulose-induced *Bifidobacterium* expansion in patients with advanced liver disease correlated with improved survival, suggesting a potential protective effect in specific clinical settings (92). Similarly, the evidence concerning *Weissella confusa* is, *nomen omen*, confusing. In a rat model of MASLD, increased abundance of *W. confusa* was associated with elevated triglyceride and cholesterol concentrations, as well as increased endogenous ethanol production (93). In contrast, a *W. confusa* Zjuids-D034 demonstrated hepatoprotective effects and improved intestinal barrier integrity in an aging mouse model (94). These apparently contradictory findings across studies highlight the importance of considering strain-level differences, as well as microbial community context rather than individual taxa alone. The impact of individual bacterial taxa may depend on their co-occurrence patterns, interactions with other members of the gut microbiota, host factors, and environmental conditions. Therefore, associations between individual bacterial taxa and MASLD progression should not be interpreted in isolation (95).

**Figure 1.**
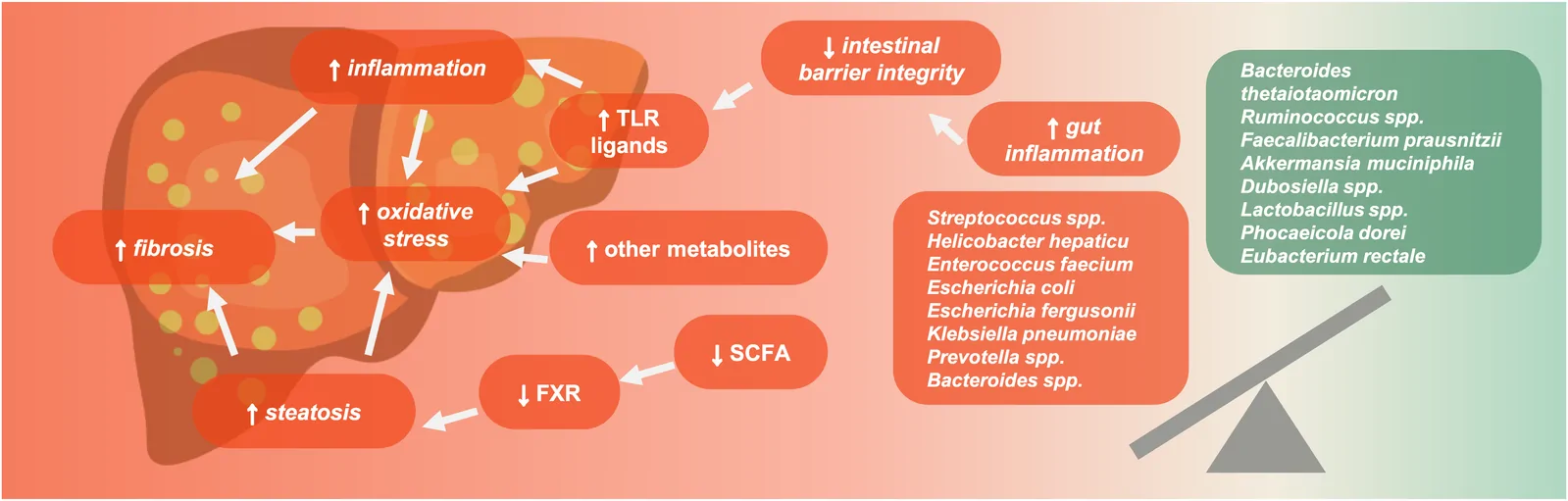
Balance between microbial species decides on MASLD progression. TLR, Toll-like receptor; SCFA, short-chain fatty acid; FXR, farnesoid X receptor.

The described associations indicate that MASLD progression is not driven by individual taxa alone but rather emerges from the balance between microbial species and their collective metabolic activity (Fig 1).

## Archaea

In addition to changes in bacterial abundance in human cohorts with MASLD and MASH, the relative abundance of the archaeon *Methanobrevibacter smithii* is also reduced (72, 96). Importantly, reduced abundance of *Methanobrevibacter*, particularly *M. smithii*, has also been identified in a prospective human cohort as a microbial marker associated with an increased risk of MASLD development (97). Archaea are in general much less studied than bacteria, and hence, our understanding of their contribution to liver diseases is limited to identification of correlative relationships.

## Gut Eukaryota

The gut eukaryome has also been implicated in MASLD, primarily based on observational studies in human cohorts. Among fungal genera, *Mucor* shows the most consistently reported association with this condition (49, 98, 99, 100).

Other fungal taxa, including *Candida albicans, Cyberlindnera jadinii, Babjeviella inositovora, Pseudopithomyces sp.* c174, *Aspergillus sp.* c25, and *Ascochyta* c213, have also been reported to show increased abundance in patients with MASLD. Furthermore, an increased abundance of *Pleosporales* has been observed in patients with MASH (97). Comparative analyses of MASLD and alcohol-associated liver disease have further demonstrated a higher abundance of *Mucor* in MASLD. Notably, these differences were already evident at early stages of the disease and became more pronounced with fibrosis progression (100). Moreover, in mice it was shown that depletion of fungi with amphotericin B resulted in reduced steatosis, fibrosis, ALT activity, and triglyceride and cholesterol levels in mouse livers, while also decreasing fecal levels of *C. albican*s, *Mucor*, and *Rhizopus spp*.

In the gut, there are also other eukaryotes in addition to fungi. However, they are rarely reported as their abundance is low, and reference databases for them are less established.

## Bacteriophages

The gut virome, an integral component of the gut microbiome, may contribute to MASLD pathogenesis by modulating bacterial community composition. Patients with MASLD exhibit a decrease in viral diversity and a lower proportion of phages, which may reflect the impaired stability of the gut ecosystem in comparison with healthy humans (101). Moreover, abundance of phages targeting *Bacteroides* is increased and abundance of phages associated with genera regarded as beneficial in the context of MASLD, such as *Faecalibacterium* and *Prevotella*, is reduced (101, 102). Beyond these observational findings, bacteriophages have been investigated experimentally as a means of modulating the gut microbiota. In a mouse model of steatohepatitis induced by high alcohol-producing *K. pneumoniae*, treatment with a *K. pneumoniae*-specific bacteriophage reduced bacterial abundance and ameliorated hepatic steatosis, liver injury, inflammation, and metabolic abnormalities (43).

## Microbiota-Associated Metabolites Impacting Liver in the Context of MASLD

Although compositional changes in the gut microbiota provide important insights, it does not fully capture the functional consequences of microbial disruption. Therefore, increasing attention has been directed toward microbial-derived metabolites (103, 104). They are considered important mediators of host–microbiota interactions and may more directly influence MASLD development and progression.

## TLR Signaling Binding Molecules

### LPS

LPS are a heterogeneous group of molecules that are key components of the outer membrane of Gram-negative bacteria, and because of their effects on humans are often referred to as endotoxin. Each LPS molecule consists of three elements: core oligosaccharide, O-polysaccharide, and lipid A, which is the primary toxic component responsible for stimulating the immune response (105).

Microbiota of MASLD patients showed increased abundance of endotoxin-producing bacteria, such as *Enterobacter cloacae* B29, *Escherichia coli* PY102, and *Klebsiella pneumoniae* A7. Evidence for their contribution to MASLD came from germ-free mice, which developed severe disease only when fed a HFD and colonized with the LPS-producing strain, whereas the LPS-producing knockout strain Δ*waaG* or only HFD-fed strain failed to elicit the same extent of disease (106).

**Figure 2.**
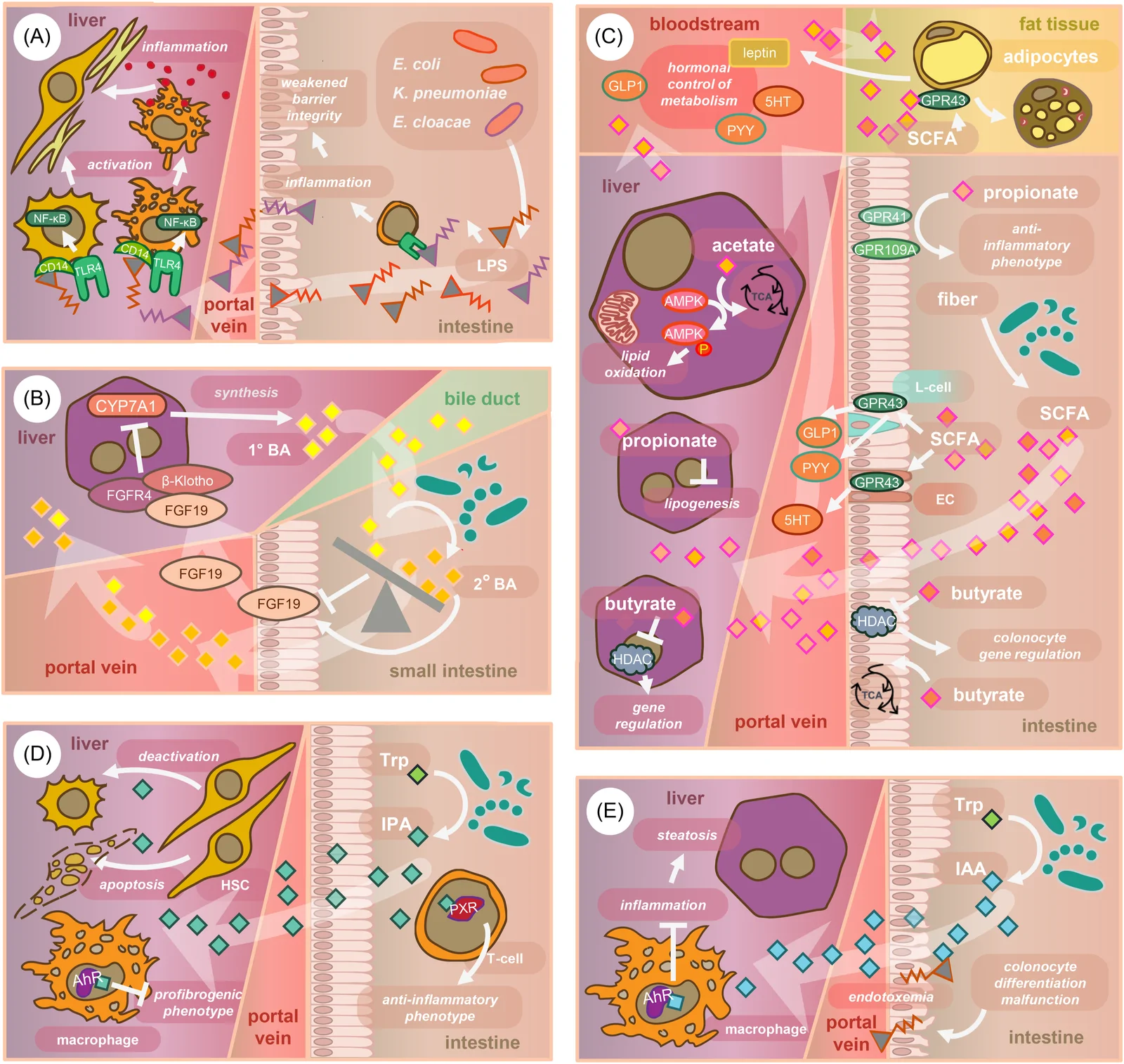
Mechanisms of metabolite action in the context of MASLD. **(A)** LPS drives multi-organ inflammation and weakens the gut integrity. **(B)** Primary and secondary BAs travel in a closed loop between the gut and liver, whereas beneficial microbiota keeps the ratio between them balanced. **(C)** SCFAs affect body metabolism, directly impacting the energy balance of the liver and gut epithelium. **(D)** IPA prevents the fibrosis progression via multicellular anti-inflammatory control. **(E)** IAA impairs the gut barrier while preventing steatosis in the liver via AhR signaling. LPS, lipopolysaccharides; 1° and 2° BA, primary and secondary bile acid; SCFA, short-chain fatty acid; EC, enterochromaffin cell; Trp, tryptophan; IPA, indole-3-propionic acid; HSC, hepatic stellate cell; IAA, indole-3-acetic acid.

As shown in several studies, in MASLD and MASH patients’ serum endotoxin levels are significantly higher than in healthy individuals (107, 108, 109). The levels of LPS in the blood negatively correlate with intestinal zonulin levels, a marker of intestinal barrier integrity (108). When the intestinal barrier is disrupted, LPS flows into the body, and because of its anatomical location, the liver is particularly exposed to it. Upon translocation to the liver, LPS binds to CD14, and then binds and activates TLR4 on hepatocytes (110), stellate cells (111), and immune cells (112, 113). TLR4 activation leads to activation of the NF-κB pathway in a dose-dependent way and triggers proinflammatory signaling cascades (108, 109). Activation of the immune system via TLR4 leads to production of proinflammatory cytokines and infiltration of immune cells into the liver (Fig 2A). The number of TLR4+ macrophages and levels of soluble CD14 correlate with LPS levels (114, 115). Moreover, disruption of TLR4 signaling, either by genetic deletion or by pharmacological inhibition, renders mice significantly less susceptible to HFD-induced MASLD than wild-type controls (106, 108). It was shown that LPS not only interacts with cell surface receptors but also was identified in the cytoplasm in hepatocytes of patients (108). Intracellular LPS acts via TLR4-independent mechanisms (109). One example is the cytosolic detection of LPS by caspase-4/5 in humans or caspase-11 in mice, leading to noncanonical inflammasome activation (116, 117). Importantly, LPS levels can be reduced in MASLD patients, and liver parameters can be improved as shown in a 12-mo intervention with a lipase inhibitor, orlistat (107).

Although the role of LPS is the most extensively studied, other pathogen-associated molecular patterns (PAMPs) also likely play a role in exacerbating inflammation in the liver in a TLR signaling–dependent manner. These include peptidoglycans (118), bacterial proteolipids, lipoteichoic acid (119), flagellin (120, 121), N-formylmethionine (122), and unmethylated CpG DNA (123, 124).

Immune response can also be induced via TLR-independent mechanisms. One such example is activation via bacterial glycolipids, which, once they cross from the intestine to the liver, act as antigens presented by CD1d molecules to NKT cells, leading to their activation and IFN-γ production. This mechanism was demonstrated by using glycolipid α-galactosylceramide in an acute hepatitis mouse model (125). However, although likely, it remains unclear whether glycolipids play a role in chronic conditions like MASLD.

### Cytolysin

In addition to PAMPs, which comprise highly conserved microbial molecules such as lipids, proteins, and nucleic acids recognized by host pattern recognition receptors (126), the microbiota can also influence the host through the production of secreted virulence factors. Cytolysins are pore-forming toxins produced by many bacterial taxa (127). Their functions include promoting bacterial dissemination, competition with other microorganisms for niche colonization, and damaging host cells. They exert their cytotoxic effects by forming β-barrel pores in the membrane of host cells, allowing ions and small molecules to pass across the membrane, disrupting osmotic balance, and leading to cell death (128).

*Enterococcus faecalis*–derived cytolysin was demonstrated to cause hepatocyte damage and worsen outcomes in alcoholic hepatitis in mice and in humans (129, 130). The amount of toxin translocating to the liver and hence the extent of liver damage depend on the degree of intestinal permeability. Then, hepatocyte damage leads to activation of the immune system: in particular, damage-associated molecular patterns released from hepatocytes, such as HMGB1, mitochondrial DNA, ATP, and histones, activate TLR-, RAGE-, and P2X7-mediated signaling (131). This mechanism may have broader implications in other liver conditions, including in MASH, which is associated with higher levels of taxa capable of cytolysis (132).

### Sphingolipids

Sphingolipids are structural components of membranes of eukaryotic cells and are synthesized de novo through a serine palmitoyltransferase–dependent pathway (133). Notably, numerous bacteria, including representatives of the genera *Bacteroides* (134) and *Prevotella* (135), possess genes encoding serine palmitoyltransferase, which enables them to synthesize sphingolipids (136).

In mouse models, colonization with *B. theta,* capable of synthesizing sphingolipids, ameliorated hepatic steatosis compared with mutants lacking this ability. Moreover, through tracking labeled bacterial lipids in vivo, it was demonstrated that *B. theta*-derived sphingolipids are absorbed and may influence hepatic lipid metabolism by affecting lipid storage and pathways involved in fatty acid β-oxidation (136, 137). In addition to direct transfer of intact lipids from the gut, bacteria could modulate the hepatic sphingolipid pool by influencing the regulation of de novo sphingolipid synthesis (137). Moreover, bacterial sphingolipids modulate host immune function in the intestine, possibly influencing gut permeability. For example, glycosphingolipids produced by *Bacteroides fragilis* modulate NKT lymphocytes (138), whereas sphingolipids produced by *B. theta* have anti-inflammatory effects in the intestine in animal models (139). It is possible that different types of sphingolipids are hepatoprotective and proinflammatory in the gut or that the same molecules have dual functions. Moreover, it is needed to disentangle the contribution of microbial and host sphingolipids to observed phenotypes. However, studies at this level of detail are not available yet.

## The Role of BAs

Although BAs are synthesized from cholesterol in hepatocytes, the diversity of this molecular class is largely generated by gut microbiota through their conversion into secondary BAs (140, 141). Hence, BAs are categorized as primary, such as cholic acid and chenodeoxycholic acid (CDCA), and secondary, that is, deoxycholic acid (DCA), lithocholic acid, ursodeoxycholic acid (UDCA), and hyodeoxycholic acid (142, 143). The transformation of primary BAs into major secondary forms, such as DCA and lithocholic acid, is mediated by the bacterial *bai* operon. This operon is found in rare members of the *Firmicutes*, most notably *Clostridium scindens* and, in murine models, *Extibacter muris* (98). In addition to their function in lipid absorption, BAs are crucial signaling molecules. Briefly, secondary BAs are reabsorbed into the liver from the gut and act as potent signaling molecules, mainly via FXR and TGR5 receptors on the liver and other organs (135, 136). Importantly, excessive accumulation of BAs leads to hepatotoxicity, inflammation, and profibrotic phenotypes in stellate cells (129, 131, 133).

Disturbances in BA metabolism contribute to the pathophysiology of metabolic diseases, including MASLD (144, 145). There is a global trend toward an increased proportion of primary, conjugated BAs to secondary BAs in the serum of MASLD and MASH patients, suggesting that the conversion of BAs by the microbiota in those patients is impaired (95, 132, 139, 140, 141, 146). This is confirmed by the correlation of levels of primary BAs, cholic acid and CDCA, in stool and MASLD severity (147, 148). In mouse models, microbiota modulation through increasing dietary fiber altered the BA profile toward increased UDCA and tauroursodeoxycholic acid and decreased DCA, which mitigated liver damage (149).

BA levels are regulated via negative feedback through FXR, inducing FGF19 synthesis in enterocytes (Fgf15 in mice). FGF19 then travels to the liver via portal blood, binds to hepatic FGFR4/β-Klotho, and triggers signaling cascades that inhibit CYP7A1 (150, 151, 152, 153). Hence, increased fecal BA loss because of microbiota dysregulation results in a compensatory increase in their de novo synthesis by CYP7A1 activity in hepatocytes (Fig 2B).

In addition to modulation of BA production, FGF19 impacts hepatocyte metabolism directly through inactivation of glycogen synthase kinase 3 through an insulin-independent mechanism (154, 155). Moreover, taurocholic acid is a BA associated with MASLD and fibrosis and has been shown to enhance triglyceride accumulation in hepatocytes under high cholesterol conditions in vitro (34, 149).

In the liver, BAs act on macrophages, including Kupffer cells, via TGR5 and FXR typically lessening inflammatory phenotypes (157, 158, 159, 160, 161). Activation of TGR5 leads to an increase in intracellular cAMP and inhibition of NF-κB signaling, thereby reducing the production of proinflammatory cytokines in macrophages and Kupffer cells (157, 158, 162). Similarly, in CD4^+^ T cells, the secondary BA isoallolithocholic acid promotes regulatory T-cell differentiation through an NR4A1-dependent mechanism (163). However, different BAs can exert distinct effects depending on macrophage populations and signaling context (164).

BAs can modulate intestinal barrier integrity through direct effects on intestinal epithelial cells and through changes in gut microbiota composition. For example, the primary BA cholic acid can impair intestinal epithelial repair by suppressing intestinal stem cell renewal, thereby exacerbating intestinal injury (165). Conversely, specific secondary BAs can also support intestinal barrier integrity. For example, taurodeoxycholic acid was shown to improve intestinal barrier function by enhancing tight junction protein expression through Nrf2 signaling (166). Moreover, alterations in BA availability can reshape the gut microbiota and its inflammatory potential, highlighting the bidirectional interplay between BAs and the intestinal ecosystem (148, 167). In addition, BAs act on metabolic homeostasis beyond the gut and liver, indirectly impacting MASLD development. For example, through TGR5 signaling, they modulate energy expenditure in adipocytes, and, through vasodilation on endothelial cells, promote glucose tolerance via GLP-1 production in the pancreas (161, 168). BAs also can cross blood–brain barrier and act on the brain directly. However, it is unclear whether there are consequences for the development of liver diseases (169). Because of the diversity of this group of compounds, the functions of many BAs remain poorly understood. For example, interesting observations concern sulfated BAs, for example, G-CDCA-3S and G-UDCA-3S, whose changes are associated with fibrosis in MASLD (34). However, it is not clear whether they contribute to the disease and how. A wide range of cell types respond to BAs via diverse receptor-mediated mechanisms. In addition to the well-characterized FXR and TGR5, BAs are sensed by other receptors, including the pregnane X receptor, vitamin D receptor, constitutive androstane receptor, and sphingosine-1-phosphate receptor 2 (170, 171, 172, 173). Many of these receptors are expressed ubiquitously across cell types. This complexity underscores the biological importance of BA signaling and the need for further research.

## Small Molecules Originating From Gut Microbiota

### Short-chain fatty acids

SCFAs are bacterial metabolites and are produced mainly in the large intestine through bacterial fermentation of plant polysaccharides (174). Although SCFAs encompass five molecules, in the gut the shorter three are dominant (acetate, propionate, and butyrate), whereas longer valerate and caproate are much less abundant and therefore less studied (56, 175, 176). The levels of SCFA in the gut depend on fiber consumption and on the amount of SCFA producers in the microbiota (89, 167). Although many bacterial taxa contribute to SCFA production, their relative contribution differs between individual SCFAs and depends on many factors, including bacterial strains, available substrates, interactions between bacteria such as cross-feeding, and SCFA metabolism by the host (177, 178, 179, 180).

Experimental studies demonstrated beneficial effects of butyrate supplementation in distinct mouse models of MASLD, including Western diet–induced MASH and ovariectomy/HFD-induced MASLD, with reductions in hepatic steatosis and fibrosis (175, 181). Similarly, dietary interventions that increase endogenous SCFA production, such as fiber supplementation, have been associated with improved metabolic and hepatic parameters (182). However, accumulating evidence indicates that the relationship between SCFAs and MASLD is more complex. In a murine model, microbiota-derived acetate generated from excess dietary fructose was transported to the liver where it became a substrate for de novo lipogenesis (183).

Circulating SCFAs showed different associations depending on the stage of disease. In MASLD, acetate levels are lower than in healthy controls, but propionate, formate, valerate, and α-methylbutyrate are higher. On the other hand, propionate, butyrate, valerate, and α-methylbutyrate were positively associated with fibrosis (176). SCFAs affect liver physiology in three major ways: (1) they act locally in the gut and improve the intestinal barrier, (2) they are absorbed into the bloodstream and impact the liver directly, and (3) they influence the function of other organs, for example, endocrine system, which in turn regulates liver function (184, 185) (Fig 2C).

Firstly, SCFAs promote intestinal barrier integrity by acting as the primary energy source for epithelial cells. Well-nourished epithelium expresses adequate levels of tight junction proteins, that is, claudins, zonulins, and occludins (175, 186). Butyrate also induces the production of mucins by goblet cells, further strengthening the barrier (187, 188, 189). Moreover, butyrate and propionate bind to GPR41 and GPR109A on the surface of epithelial cells, inducing intracellular signaling pathways leading to anti-inflammatory responses. SCFA receptors GPR41, GPR43, and GPR109A are also present on immune cells in the gut and modulate immune responses through NF-κB (190, 191, 192). In addition to acting via receptors, butyrate is also a histone deacetylase inhibitor (193) and through this mechanism regulates gene expression not only in colonocytes, but also in immune cells (194, 195).

Secondly, SCFAs impact the host systemically, for instance, by acting on the brain through stimulation of the vagus nerve or by inducing hormonal responses (196, 197, 198). SCFAs via the GPR43 receptor induce secretion of GLP-1 (199) and peptide YY by enteroendocrine L cells (200) and production of serotonin by the gut enterochromaffin cells, influencing the metabolic state of the body (201). In adipose tissue via GPR43 signaling, SCFAs promote browning of the adipose tissue and leptin production by adipocytes, promoting energy burning instead of storage (197, 202). SCFAs also stimulate other organs and a plethora of metabolic pathways, which were extensively reviewed elsewhere (203, 204).

Finally, SCFAs act directly on hepatocytes. Acetate induces fatty acid oxidation through activating adenosine monophosphate–activated protein kinase, thereby promoting energy expenditure and fat burning in the liver, whereas propionate limits lipogenesis, which leads to a decrease in intrahepatic triglyceride concentrations (54, 173). Butyrate promotes hepatic GLP-1R expression, sensitizing them to GLP-1, which leads to a reduction in lipid accumulation (202, 205, 206). In the liver, SCFAs also have a broadly anti-inflammatory influence on the immune compartment, including macrophages, Kupffer cells, and neutrophils (206, 207, 208). Acetate and propionate promote regulatory phenotype in CD4^+^ T cells (209), and butyrate enhances generation of memory CD8^+^ T cells (210). Although typically SCFAs have anti-inflammatory and proregulatory functions, in CD8^+^ T cells, the key regulators of injury in the context of MASLD (211, 212), acetate promotes an autoaggressive phenotype (213).

### Branched-chain fatty acids

Branched-chain fatty acids (e.g., isobutyrate and isovalerate) are a class of SCFAs produced in the intestine by proteolytic fermentation of BCAAs (55, 200). The abundance of BCAAs, as well as of BCAA producers, is significantly promoted by high protein intake, whereas the presence of fermentable carbohydrates inhibits their activity (214, 215). Unlike SCFAs, branched-chain fatty acids are higher in the blood of patients with MASLD and MASH in comparison with healthy individuals. This is accompanied by higher expression levels of genes related to their metabolism in the liver (*BCAT1*, *BCAT2, BCKDHA*) (56, 200). It is unclear what the consequences of these differences are. To date, studies are limited to in vitro assays, which present contradictory results. In hepatoma cells, isobutyrate and isovalerate activate mTORC1/S6K1 pathway and promote hepatic gluconeogenesis (216). Another in vitro study shows that branched-chain fatty acids inhibit both lipolysis and de novo lipogenesis in primary adipocytes (217). However, in other contexts, experiments in animal models suggest a potential anti-inflammatory role (218, 219).

### Tryptophan derivatives

Indole and its derivatives are metabolites of the amino acid tryptophan. Some, including serotonin and melatonin, are synthesized by the host.

Gut microbiota metabolizes tryptophan through several pathways, which are absent in humans leading to the formation of indole and related metabolites. Indole is produced directly from tryptophan by bacterial tryptophanase, whereas other indole derivatives, including indole-3-propionic acid (IPA), indole-3-acetic acid (IAA), and indole-3-aldehyde, are generated through distinct microbial pathways, including those involving indole-3-pyruvate, indole-3-lactate, and indole-3-acetaldehyde (220, 221). A wide range of bacterial taxa, including *Bacteroides*, *Lactobacillus*, and *Clostridium*, generate a diverse range of indole metabolites, several of which have been implicated in MASLD and are discussed in the following sections.

Importantly, some indole derivatives function as plant hormones, and their concentrations are often very high (in the microgram per gram) in plants (222). Hence, the contribution to the levels of indole derivatives in the gut may vary widely depending on individual dietary preferences in addition to microbiota composition.

### Indole

Higher levels of indole are associated with lower inflammation and lower liver fat accumulation in humans with MASLD. Supplementation of indole to HFD-fed mice leads to amelioration of hepatic steatosis and reduction of aminotransferase activity (223, 224). There are several mechanisms in play; indole improves the intestinal barrier (225, 226), impacts metabolism systemically via promoting incretin secretion by enteroendocrine L cells (227), acts on hepatocytes directly via aryl hydrocarbon receptor (AhR), and suppresses macrophage proinflammatory activation (224).

### Indole-3-propionic acid

Levels of IPA are significantly reduced in the stool and serum of MASLD patients compared with the healthy control group (90). Similarly, in mouse models of MASLD, IPA was lower than in control animals (156, 228) and IPA supplementation led to reduced steatosis, lower inflammatory infiltration, and a decrease in lipogenesis gene expression (90, 149). Moreover, IPA has antifibrotic activity, as it acts directly on hepatic stellate cells, leading to a reduction in the expression of extracellular matrix components, including collagens (228). In vitro, IPA exerted proapoptotic effects in the hepatic stellate cell line LX-2, suggesting that its antifibrotic activity may involve both reversal of the profibrotic phenotype of hepatic stellate cells and induction of their apoptosis (229). IPA may also affect hepatic fibrosis indirectly by targeting macrophages and, through AhR signaling, inhibiting their profibrogenic phenotype (Fig 2D) (230). Beyond its effects on the liver, IPA also acts in the intestine, where it promotes intestinal barrier integrity through activation of pregnane X receptor, modulates T-cell metabolism, and influences the composition and function of the gut microbiota (228, 231, 232), as well as host metabolism (233).

### Indole-3-acetic acid

Similar to IPA, levels of IAA are lower in patients with MASLD (234) and in mouse models of MASLD (235, 236). Oral supplementation of IAA results in a reduction of steatosis, inflammation, and fibrosis in mice with diet-induced MASLD generated using the Western diet–based DIAMOND model (236). Mechanistically, IAA attenuates a proinflammatory phenotype in macrophages via AhR signaling (235, 236). Moreover, IAA supplementation leads to an AhR-dependent reduction in free fatty acid accumulation in hepatocytes, accompanied by down-regulation of fatty acid synthase (*Fasn*) and SREBP-1c expression and improved mitochondrial respiration (235, 237). However, IAA was also linked to the energy imbalance of intestinal stem cells and defects in gut epithelium secretory lineage commitment. Such disruptions in the intestinal barrier usually have a proinflammatory effect on the liver, because of PAMP translocation (Fig 2E) (238, 239). Further cytotoxic effects of IAA were reported to be linked to cells with a high concentration of myeloperoxidase, such as neutrophils. Mechanistically, myeloperoxidase can oxidize IAA into toxic products such as 3-methylene-2-oxindole, leading to cell death (240, 241).

### Other indoles

Indoles constitute a large and heterogeneous group, most of whose members remain insufficiently characterized. However, some emerging findings are beginning to point to new directions. For example, two-stage Mendelian randomization analysis demonstrated that increased 3-indoleglyoxylic acid levels were associated with a lower likelihood of developing the MASLD, yet the mechanisms are unknown (242). Other indoles were studied in the context of intestinal homeostasis. Indole-3 lactic acid promotes tolerogenic intraepithelial CD4^+^CD8αα^+^ double-positive T cells (243). Indole 3-aldehyde induces the expression of IL-22 in intestinal lymphoid cells via an AhR-dependent mechanism, also leading to anti-inflammatory effects (244, 245). In addition, higher levels of indole-3-aldehyde have been associated with better outcomes in MASLD (246).

### Trimethylamine N-oxide

Although trimethylamine N-oxide (TMAO) is produced by the host, its levels are dependent on bacteria-derived precursor trimethylamine (TMA) (247, 248). Although TMAO was first studied in the context of cardiovascular diseases (249), it has also been implicated in other conditions, such as MASLD (250, 251). Increased levels of TMAO in blood correlate with higher abundance of TMA-producing genes in microbiomes from MASLD patients compared with the healthy cohort (89). Orthologs and homologs of enzymes producing TMA such as CntA/CntB and YeaW/YeaX have been identified in a wide range of gut microorganisms (252). Experiments in HFD-fed mice showed that supplementation of TMAO exacerbated steatosis and elevated AST levels (253).

Mechanistically, TMAO is suggested to bind to the endoplasmic reticulum stress kinase PERK in hepatocytes, leading to FoxO1-driven changes in gene expression. This results in metabolic dysfunction and dysregulated BA metabolism (253, 254). Moreover, TMAO may contribute to MASLD through the activation of immune cells toward proinflammation (255) and affects metabolism through pancreatic β-cells and adipocytes (256, 257).

### 5-Aminovaleric acid betaine

5-Aminovaleric acid betaine (5-AVAB), also known as trimethyl-5-aminovaleric acid or δ-valerobetaine (258, 259), is a gut microbiota–dependent metabolite derived from trimethyllysine through action of lysine 2-monooxygenase and 5-aminovaleramidase (260). Specific gut bacteria, including *Bifidobacteriaceae* and *Coriobacteriaceae* families (261, 262) and also *E. faecalis* and *Pseudomonas aeruginosa*, have been identified as key producers of this metabolite (263). Similar to TMAO, it was first studied in the context of cardiovascular diseases (263, 264). However, it was later also identified to be elevated in patients with MASLD (261, 265, 266, 267). According to clinical data in human cohorts, 5-AVAB levels independently predict the risk of developing glucose metabolism–related disorders and type 2 diabetes, while also correlating with markers of fatty liver disease. Moreover, in individuals not treated with antidiabetic medications, 5-AVAB levels serve as a predictor of future fat accumulation in the liver over the long term (268). In mice, 5-AVAB administration exacerbates diet-dependent fatty liver disease (261, 265). Because of its structural similarity to γ-butyrobetaine, it is suggested that 5-AVAB inhibits γ-butyrobetaine hydroxylase, a key enzyme in carnitine synthesis. Reduction in the levels of carnitine leads to fatty acid oxidation impairment, causing lipid and long-chain acetyl-CoA accumulation and metabolic stress in the liver (261, 265).

### L-phenylalanine

L-phenylalanine (L-Phe) is an essential amino acid obtained primarily from the diet, whose metabolism is also influenced by the gut microbiota. Circulating L-Phe levels are elevated in patients with MASLD. In mouse models, chronic exposure to high levels of L-Phe led to induction of hepatic steatosis associated with increased de novo lipogenesis and inhibition of fatty acid β-oxidation. It is suggested that impaired mitophagy plays a role in this process; however, more research is needed to fully understand the molecular underpinnings (269)

### L-aspartate

The amino acid L-Asp has been suggested to have a protective role in metabolic liver diseases, including MASLD and MASH (270, 271). Although L-Asp is a nonessential amino acid, its levels can be affected by the microbiota. Specifically, supplementation with *A. muciniphila* led to a significant increase in L-Asp levels in feces, plasma, and liver in mice (68). Moreover, this intervention also led to increased levels of L-Asp transporter in the small intestine, suggesting increased absorption in the small intestine (68). The beneficial effects of L-Asp appear to involve both direct and indirect mechanisms. In hepatocytes, L-Asp promotes lipid oxidation through activation of the LKB1–adenosine monophosphate–activated protein kinase axis, improves mitochondrial function, and reduces oxidative stress (69, 272). In addition, L-Asp may act indirectly through platelets by increasing platelet cGMP levels and suppressing platelet activation and ATP release, thereby limiting ATP-mediated mitochondrial fragmentation and lipid accumulation in hepatocytes (270). L-Asp has also been reported to improve hepatic mitochondrial function and attenuate liver fibrosis (273).

### L-citrulline

L-citrulline (L-Cit) is a nonessential amino acid, an intermediate in the urea cycle, and also a by-product of nitric oxide synthesis from L-arginine in kidneys (274, 275). The gut microbiome of patients with MASH harbors a reduced abundance of genes involved in L-Cit biosynthesis, and this reduction correlates with the abundance of *Parabacteroides distasonis*. Decreased L-Cit potentially could contribute to ammonia accumulation typically observed in cirrhotic patients (29). Supplementation of L-Cit in animal models of MASLD improves glucose and lipid metabolism, including fasting blood glucose, insulin resistance, and triglyceride levels (276, 277, 278). In addition to its role in enhancing the urea cycle and inhibiting lipogenesis, it was proposed that L-Cit also functions through improving intestinal barrier integrity (276).

### Phenylethylamine

PEA is a derivative of L-Phe synthesized by neurons in humans, and it was linked to hepatic encephalopathy (279, 280, 281). Notably, it can be produced by members of the gut microbiota, which may be more relevant in the context of liver diseases than the host-dependent synthesis. Interestingly, PEA was shown to be produced through a cross-feeding mechanism in which *B. theta* produces L-Phe, which is subsequently used by *Morganella morganii* or *Ruminococcus gnavus* for its conversion to PEA. Gut microbiota–derived phenylethylamine impairs insulin sensitivity in metabolic syndrome and irritable bowel syndrome (282, 283). PEA is a highly neuroactive molecule, which acts via trace amine-associated receptor 1, leading to neurotransmitter release modulation; however, it is not clear whether this mechanism is linked to its action in the context of the liver (284).

### Histidine and histamine

Histamine is normally produced by the host and also can be supplied by microbiota (285). In the gut, producers that carry the histidine decarboxylase gene, such as members of *Bacteroides*, *Clostridium*, *Bifidobacterium*, *Fusobacterium,* and *Lactobacillus*, convert L-histidine to histamine (282, 285). Histamine performs key distinct functions in the human body as it participates in immune response, triggers gastric secretion, and regulates the sleep–wake cycles (286). Patients with MASLD have an increased number of bacterial histidine utilization system genes responsible for the catabolism of histidine and lower plasma levels of this molecule. A decrease in histidine levels results in intensification of de novo lipogenesis and an increase in inflammation. Histidine supplementation in mouse models of MASLD led to alleviation of steatosis (287). This suggests that the balance between levels of histidine and histamine in the gut is physiologically relevant in the context of liver disease. However, the contribution of the host and microbiota to the maintenance of this balance, as well as specific metabolic pathways affected, warrants further investigation.

### Hippurate

Similar to TMAO, hippurate is a host–microbiome cometabolite. It is formed by the liver through the conjugation of glycine with benzoate, which comes from microbial degradation of dietary polyphenols. Microbiome analyses have shown that plasma hippurate concentration is positively associated with the *Ruminococcaceae* and *Rikenellaceae* families, while being negatively associated with the *Lachnospiraceae family*. Specifically, positive relationships are observed with the presence of the species *F. prausnitzii* and members of the order *Clostridiales*, whereas negative correlations involve the genera *Eubacterium* and *Ruminococcus*. Because of these associations, hippurate is recognized as an important metabolic marker of overall gut microbiome diversity (288). In humans, higher blood hippurate concentrations have been consistently associated with a favorable metabolic profile (288, 289, 290). Similarly, in both plasma and liver tissue hippurate concentrations are negatively correlated with the severity of MASLD (291). In vitro, hippurate treatment lowered triglyceride accumulation and glucose uptake in hepatocytes (291). Yet, to our knowledge, in vivo studies establishing causation are still lacking.

### Succinate

Succinate, a Krebs cycle intermediate, is secreted in the gut by bacteria of the *Bacteroidaceae* family. Succinate is recognized as a potent signaling molecule acting on the succinate receptor 1 (292). Increased circulating succinate concentration was observed in metabolic disorders, including MASLD (293, 294, 295). Succinate is generally thought to promote steatosis. Notably, in succinate receptor 1–deficient mice, increased hepatic steatosis and de novo lipogenesis were observed (296, 297). Succinate stimulates the activation of hepatic stellate cells and production of α-smooth muscle actin and type I collagen, as well as proinflammatory cytokines (298, 299). Importantly, it is unclear to what extent succinate derived from the gut contributes to these processes.

## Conclusions

**Figure 3.**
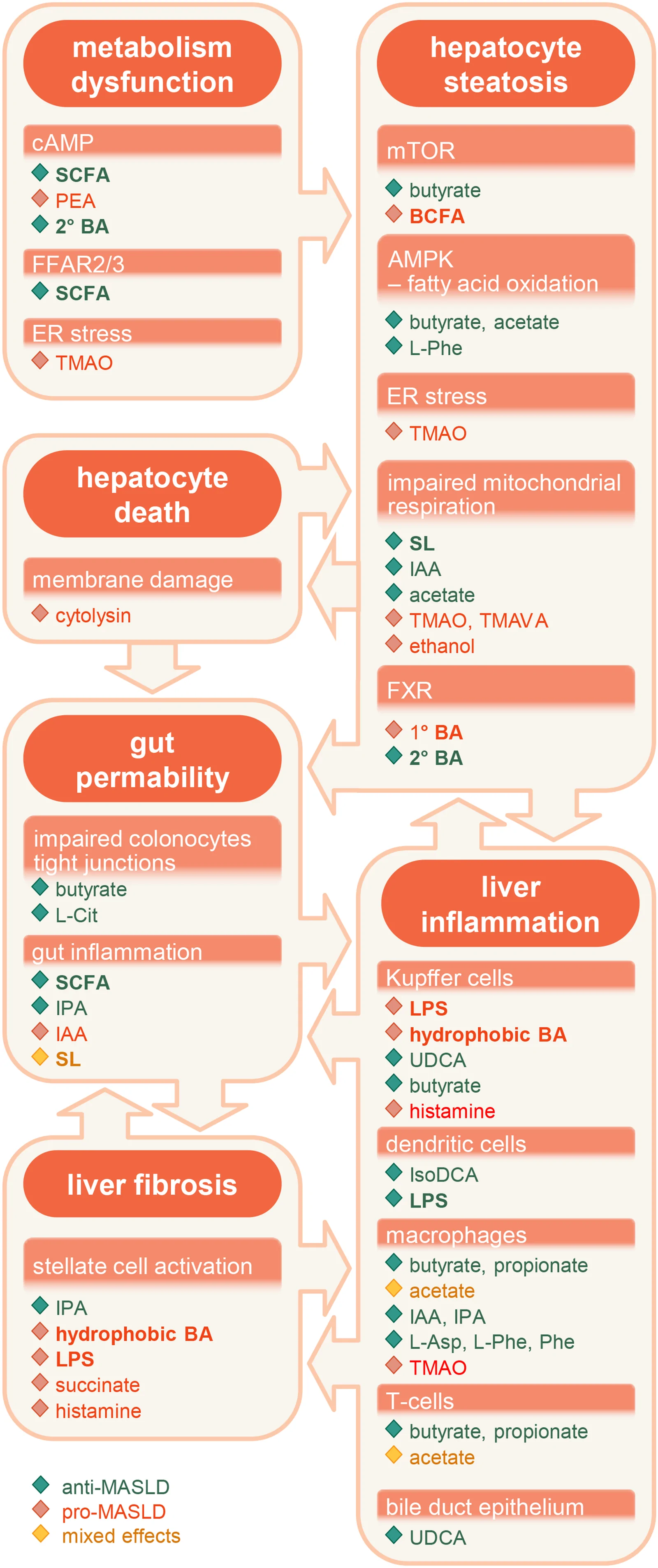
Metabolites’ contribution to MASLD development. SCFA, short-chain fatty acid; PEA, phenylethylamine; 1° and 2° BA, primary and secondary bile acid; ER, endoplasmic reticulum; TMAO, trimethylamine N-oxide; L-Cit, L-citrulline; IPA, indole-3-propionic acid; IAA, indole-3-acetic acid; SL, sphingolipid; LPS, lipopolysaccharides; BCFAs, branched-chain fatty acids; TMAVA, trimethyl-5-aminovaleric acid; UDCA, ursodeoxycholic acid; IsoDCA, 3β-hydroxy deoxycholic acid; L-Asp, L-aspartate; Phe, phenylalanine.

The reviewed literature strongly indicates that intestinal bacteria and their metabolites constitute a crucial element of the gut–liver axis and may modulate the course of liver diseases, in particular, MASLD. It is important to emphasize that observed relationships are often correlational, and causality is usually established only in animal models. Furthermore, the complexity of the microbiota ecosystem, encompassing hundreds of species and their metabolic interactions, often makes it difficult to clearly attribute effects to specific taxa. It is worth noting that the relationships between microbiota changes and MASLD are likely bidirectional and are affected by diet and environmental factors leading to high interindividual variability (Fig 3). This means that the generalizability of results is often limited and their translation into clinical practice requires personalized approaches.

In addition to diet and lifestyle changes, modulation of microbiota (e.g., diet or probiotics) is a promising therapeutic direction. However, it requires further interventional studies considering MASLD phenotypes and functional analysis of the microbiome at the strain level. Interventions into microbiota are challenging, as probiotics often do not colonize the gut because of colonization resistance, and targeted bacterial removal with phages is at the early stages of development. Hence, instead of microbiota composition themselves, shifting the focus to microbial metabolites may be a more feasible therapeutic target. Such metabolites could be either supplemented or depleted by enzyme therapy in a similar way lactose is degraded by β-galactosidases (300). Moreover, interventions targeting improvement of intestinal permeability, treatment with anti-inflammatory agents, and treatment with LPS or ethanol synthesis inhibitors may also improve disease outcomes. Because of differences in microbiota composition and function between humans, personalized therapies based on predominant mechanisms driving disease in particular individual would likely be most beneficial.

## Supplementary Material

Reviewer comments
