# Supplementary material for: Gut microbiota shaping fatty liver disease progression
Source: Life Sci Alliance. 2026 Oct 12;10(1):e202603756. doi: 10.26508/lsa.202603756 (PMC13658947; doi:10.26508/lsa.202603756)
Supplement: Supplementary file 1 [file LSA-2026-03756_review_history.pdf]

Gut microbiota shaping fatty liver disease progression

Martyna Wysokińska, Marta Mysza, Aleksandra Uryga, and Aleksandra Kolodziejczyk
DOI: https://doi.org/10.26508/lsa.202603756

Corresponding author(s): Aleksandra Kolodziejczyk, International Institute of Molecular and Cell Biology

Review Timeline:

Submission Date:	2026-05-04
Editorial Decision:	2026-05-06
Revision Received:	2026-05-07
Editorial Decision:	2026-07-28
Revision Received:	2026-09-03
Editorial Decision:	2026-09-23
Revision Received:	2026-09-29
Accepted:	2026-09-29

Scientific Editor: Tim Fessenden

Transaction Report:

(Note: With the exception of the correction of typographical or spelling errors that could be a source of ambiguity, letters and reports are not edited. The original formatting of letters and referee reports may not be reflected in this compilation.)

May 6, 2026

Re: Life Science Alliance manuscript #LSA-2026-03756-T

Prof. Aleksandra A Kolodziejczyk
International Institute of Molecular and Cell Biology
Laboratory of Cellular Genomics
POLAND

Dear Dr. Kolodziejczyk,

Thank you for submitting your presubmission inquiry concerning a Review entitled "Microbiota and their metabolites in MASLD pathogenesis" to Life Science Alliance.

In agreement with our offer conveyed to you by another journal, we are pleased to invite submission of the full review. Please log into the system to submit your review, and contact the journal should you have any question.

Thank you for proposing this interesting contribution to Life Science Alliance. We are looking forward to receiving your review article.

Sincerely,

Tim Fessenden
Monitoring Editor
Life Science Alliance

July 28, 2026

Re: Life Science Alliance manuscript #LSA-2026-03756-TR

Dr. Aleksandra A Kolodziejczyk
International Institute of Molecular and Cell Biology
Laboratory of Cellular Genomics
Ksiecia Trojdena 4
Warsaw 02-109
Poland

Dear Dr. Kolodziejczyk,

Thank you for submitting your review article entitled "Gut microbiota shaping fatty liver disease progression" to Life Science Alliance. The manuscript was assessed by expert reviewers, whose comments are appended to this letter. We appreciate your patience while we solicited reviewer input and we regret the delay in the review process.

As you will see all reviewers remarked on the importance of this topic and appreciated the broad approach to synthesize the current state of knowledge to relate diverse organisms and metabolites that reside in the gut with fatty liver disease. All reviewers made requests to be incorporated in a suitably revised manuscript. In particular Reviewers 1 and 2 sought important corrections and clarifications and all their their comments must be addressed. While we appreciate the detailed and thorough approach of Reviewer 3, we note that all manuscripts would undergo copy editing upon acceptance at LSA. We invite you to consider their several suggestions to improve the text however you see fit. We draw your attention to their points 2, 5, 9, 46, 49, 50, 64, 91, and 112 which we concur should be addressed in some form. Responses to the other points from this reviewer are not required in a rebuttal letter.

I would be happy to discuss the revision in more detail via email or phone/videoconferencing. Please let me know which option you prefer, if any.

To upload the revised version of your manuscript, please log in to your account: https://lsa.msubmit.net/cgi-bin/main.plex

You will be guided to complete the submission of your revised manuscript and to fill in all necessary information. Please get in touch in case you do not know or remember your login name.

The typical timeframe for revisions is three months. Please note that papers are generally considered through only one revision cycle, so strong support from the referees on the revised version is needed for acceptance.

While you are revising your manuscript, please also attend to the below editorial points to help expedite the publication of your manuscript. Please direct any editorial questions to the journal office. When submitting the revision, please include a letter addressing the reviewers' comments point by point.

Thank you for this interesting contribution to Life Science Alliance. We hope that the comments below will prove constructive as your work progresses, and we are looking forward to receiving your revised manuscript.

Sincerely,

Tim Fessenden
Scientific Editor
Life Science Alliance

A. THESE ITEMS ARE REQUIRED FOR REVISIONS

-- A letter addressing the reviewers' comments point by point.

-- An editable version of the final text (.DOC or .DOCX) is needed for copyediting (no PDFs).

-- High-resolution figure, supplementary figure and video files uploaded as individual files: See our detailed guidelines for preparing your production-ready images, https://www.life-science-alliance.org/authors

-- Summary blurb (enter in submission system): A short text summarizing in a single sentence the study (max. 200 characters including spaces). This text is used in conjunction with the titles of papers, hence should be informative and complementary to the title and running title. It should describe the context and significance of the findings for a general readership; it should be written in the present tense and refer to the work in the third person. Author names should not be mentioned.

-- By submitting a revision, you attest that you are aware of our payment policies found here: https://www.life-science-alliance.org/copyright-license-fee
Please note that your manuscript is exempt from our article processing fee as it was an invited review.

B. MANUSCRIPT ORGANIZATION AND FORMATTING:

Full guidelines are available on our Instructions for Authors page, https://www.life-science-alliance.org/authors

We encourage our authors to provide original source data, particularly uncropped/-processed electrophoretic blots and spreadsheets for the main figures of the manuscript. If you would like to add source data, we would welcome one PDF/Excel-file per figure for this information. These files will be linked as supplementary "Source Data" files.

IMPORTANT: It is Life Science Alliance policy that if requested, original data images must be made available. Failure to provide original images upon request will result in unavoidable delays in publication. Please ensure that you have access to all original microscopy and blot data images before submitting your revision.

Reviewer #1 (Comments to the Authors (Required)):

This manuscript provides a review of how specific gut microbiota impacts fatty liver disease progression with a specific focus on bacterial genera/species and importantly, brief overviews of archaea, eukaryota and bacteriophages. The review also provides explanations on how microbial elements (TLR signalling molecules, bile acids and small molecules) may impact the liver in fatty liver disease. While the review is well-written, and covers a broad range of bacterial species and microbial metabolites, several issues regarding the ambiguity of the claims and the citations should be addressed.

Major comments:
1. Lines 101-104: The role of B. stercoris is discussed. In its current form, it appears that this finding is from both references (ref) [24] and [25]. However, it is only the study in ref [25], where mouse experiments were performed and subsequent hypothesis regarding B.stercoris driving pathogenesis through LPS and BCAA. Ref [24] only mentions elevated B.stercoris in MASLD human cohort and its positive correlation to BA metabolism species. This should be rectified in the manuscript.

2. Lines 116-117: The abundance of Prevotella copri has been associated with fibrosis in MASH in four independent cohorts. While these are indeed independent cohorts, 3 out of 4 are either primarily [29,30] or predominantly hispanic [31]. In ref [32], while not predominantly hispanic, the information about ethnicity was not corrected for as a confounding factor. Therefore there are 2 points to clarify:

a) Strain specificity: It is worthwhile to have a look at this review on P. copri here: https://doi.org/10.1080/19490976.2023.2249152, so as to understand the strain specific effects of this bacteria.

b) Ethnicity and diet composition may influence gut microbial composition as well as any bacterium's phenotype, as has been shown by numerous papers before. In the absence of references to studies that have looked beyond hispanic populations, it may not be prudent to assert the association of P. copri to fibrosis. The authors should provide references to such studies to support the assertions. If such studies do not exist, then this aspect of soft association of ethnicity to P. copri - fibrosis should ideally be acknowledged in the review.

3. Lines 261-262: "Faecalibacterium, Subdoligranulum, Haemophilus, and Roseburia
were positively correlated with MASLD". Contrasting observation, given the prior section on Faecalibacterium in the review. The authors should state here a bit more about contrasting observations. On further reading of the reference [53], this sentence appears to be misframed. These 4 genera are in fact negatively associated with MASLD. Therefore, the concerned sentence should be amended.

4. Lines 276-291: The contrasting roles of Eubacterium, Agathabacter and Bifidobacterium have been discussed. This maybe due to co-occurrence in microbial communities. It has been shown by several studies know singular bacterial strains cannot always alone drive gut microbial homeostasis, rather it is the communities that do so. While the authors have acknowledged this fact in one sentence at the end of the section, this aspect deserves a brief overview in the context of MASLD.

5. Lines 367-371: Section on cytolysins is missing citations.

6. Line 368: I would argue that the main function of cytolysins is not only damaging host cells, but also dissemination and competition with other microbes for niche colonization. Source: https://doi.org/10.1099/mic.0.001154

Minor comments:
1. Line 87: The authors state that they "systematically discuss taxa with the most evidence", however no criteria are provided for how evidence was evaluated or how taxa were selected for inclusion. The authors should either define their selection criteria explicitly, or reframe this as a narrative synthesis reflecting the authors' expert judgement rather than implying a systematic process.

2. Couple of grammatical errors:
Line 21: "..we put particular emphasis on.."
Line 368: "Particularly, they produce factors, whose main function is to damage host cells."
Lines 598-599: "L-Cit is a non-essential amino acid, an intermediate in urea cycle and also by-product of nitric oxide synthesis from L-arginine in kidneys."

Reviewer #2 (Comments to the Authors (Required)):

General evaluation
This review addresses a timely and important topic: the contribution of the gut microbiota and microbiota-derived metabolites to the development and progression of metabolic dysfunction-associated steatotic liver disease (MASLD). The manuscript covers a broad range of microbial components, including bacteria, archaea, fungi, bacteriophages, microbial structural products, bile acids, short-chain fatty acids, amino-acid derivatives, and other metabolites. This breadth is particularly valuable. In my opinion, the manuscript requires only minor revisions.
Minor comments
• Throughout the manuscript, it is not always clear whether the reported findings derive from human cohorts, animal models, or in vitro experiments. The authors should specify the experimental context more consistently and clearly distinguish observational associations in patients from causal or mechanistic evidence obtained in experimental models.
• The manuscript states that supplementation with Lactobacillus acidophilus for 14 days reduced circulating liver-enzyme activity in a small human cohort and cites reference 75. However, reference 75 is Wu et al., "Lactobacillus acidophilus ameliorates cholestatic liver injury through inhibiting bile acid synthesis and promoting bile acid excretion," which does not describe a 14-day human MASLD intervention. Please correct this citation and verify that the other references accurately support the corresponding statements.
• The SCFA section presents these metabolites as broadly protective, whereas their effects appear context-dependent and vary according to the individual SCFA and the biological compartment measured. For example, circulating propionate and butyrate were positively associated with significant fibrosis in a biopsy-characterized MASLD cohort cited by the authors (Thing et al., 2024). In addition, microbiota-derived acetate can support hepatic de novo lipogenesis under high-fructose conditions (Zhao et al., 2020). Please nuance this section accordingly.
• Bacteroides is repeatedly misspelled as "Bacterioides" (lines 89, 94, and 109).
• Line 329: LPS should be described more precisely as a component of the outer membrane of Gram-negative bacteria rather than generically as a bacterial "cell-wall" component.
• Line 370: "b-barrel" should be replaced with "β-barrel."

Reviewer #3 (Comments to the Authors (Required)):

The manuscript by Wysokinska et al provides a review on the contribution of the gut environment in shaping fatty liver diseases. In particular, the authors focus on metabolic-associated steatotic liver disease (MASLD) and related diseases. The current evidence provided are mostly associative, which are limited by study design in nature. Despite the comprehensive body of work that highlights a clear association between the gut environment and MASLD, the manuscript lacks key details and would benefit from multiple rounds of formatting and cleaning up in writing style (lengthy sentences) and grammar to improve readability.

Major and minor comments:
1. Line 21: typo: "we particular"
2. Line 68: It seems that gut microbiome fits better for this review than gut microbiota since gut microbiome encompasses is the collection of microorganisms and their genes, whereas microbiota limits to the living microorganisms.
3. Line 100: is this really enzyme activity or abundance?
4. Lines 102-104: Would benefit from rephrasing.

5. Lines 106-107: specify which "desirable features in in vitro assays"
6. Line 114: Is folic acid biosynthesis really upregulated or is this at the pathway level from microbial gene abundance.
7. Line 118: "in some studies" - specify.
8. Line 119 and onward: overuse of "interestingly" and "importantly". If something is interesting or important, specify why. I counted 14 "interestingly" and 10 "importantly". Overuse of "Moreover"
9. Line 124: Would benefit from describing ethanol consumption in exacerbating MASLD. The authors state the carryover of NAFLD to MASLD in terminology. NAFLD does not include alcoholic fatty liver disease or fatty liver disease from excessive alcohol consumption.
10. Line 131: Does R. farcis metabolize primary bile acids? In addition, specify how secondary bile acids influence inflammatory and fibrotic processes in the liver.
11. Line 134-135: "improved MASLD parameters" - specify.
12. Line 146: Does HiAlc-Kpn correlate to circulating and hepatic ethanol levels?
13. Lines 148-151: Would benefit from rewriting. Was there increased ethanol detected in the liver?
14. Line 155: Give examples.
15. Line 166: The comparison is between obesity and MASH. What about non-MASLD (healthy controls) vs MASLD or healthy controls vs MASH?
16. Lines 167-169: Would benefit from rewriting.
17. Lines 173-174: Can split in 2 sentences.
18. Lines 183-186: Would benefit from rewriting.
19. Lines 192-193: Would benefit from rewriting.
20. Line 209: "Amuc_1100 interacts with TLR2" - specify what the authors mean by interact. Does Amuc_1100 bind to TLR2 or indirectly regulates TLR2 through another mechanism?
21. Line 221-223: Would benefit from rewriting.
22. Line 226: Spell out FGF2.
23. Line 231: "Because they are used as probiotics" -> something like: As Lactobacillus sp are currently classified as probiotics.
24. Line 232: "In a small human cohort" - specify how many individuals.
25. Line 233: related to reference 75, in what condition are the authors referring to?
26. Line 239: "reduction of hepatocyte necrosis" -> "reduction in hepatocyte necrosis"
27. Line 249-251: Rewrite.
28. Line 251: How was Helicobacter hepaticus introduced? Through oral gavage?
29. Line 252-255: Would benefit from rewriting.
30. Line 255-257: Would benefit from rewriting.
31. Line 256: What is the sample size?
32. Line 261: In the previous section, the authors state Faecalibacterium is negatively correlated with MASLD.
33. Line 264 and in all sections: via does not need to be italicized.
34. Line 264: ...via their proinflammatory potential -> something like via their contribution in creating proinflammatory environment. Give examples.
35. Line 279: However, in the cohort -> However in a cohort.
36. Line 279: What is the sample size?
37. Line 284: What do the authors mean by depleted? Go to 0 or decreased?
38. Lines 286-289: Would benefit from rewriting.
39. Line 292: make consistent headers. The first header was bacteria associated with MASLD with specific examples of bacteria. Archea and others should have similar headers.
40. Line 300-301: Rewrite.
41. Line 302: lowercase "Increased"
42. Line 303: Are the authors referring to MASLD for "the disease"? It seems like there is overuse of pronouns (e.g., it) and unclear reference to the exact context.
43. Line 304-306: Would benefit from rewriting.
44. Line 305: Spell out ALD
45. Line 308: "in the livers in mice" -> "mouse livers"
46. Line 308-309: why is this important?
47. Lines 314-315: Would benefit from rewriting.
48. Line 322: Microbial elements -> microbiota-associated metabolites
49. Lines 323-325: Should highlight this in the introduction. The first half heavily focuses on microbial abundance associated with MASLD and the latter half focuses on functional outputs of gut microbes. The manuscript would benefit from clear distinction of these in the introduction/abstract.
50. The second section suffers from the lack of key information involving microbial pathways. What microbes are involved in the metabolic pathways of metabolite production and how are their abundances altered MASLD?
51. Line 238: Spell out LPS
52. Line 333: "Microbiota of MASLD patients contain bacteria that are high-endotoxic producers" -> "Microbiota of MASLD patients showed increased abundance of endotoxic producing bacteria"
53. Line 334-335: How was causality determined?
54. Line 341: When "the" intestinal barrier is disrupted,
55. Line 343: "Once inside the liver LPS binds to TLR4" -> Upon translocation to the liver, LPS binds to TLR4

56. Line 343: what do the authors mean by "adaptor"
57. Line 345: remove "of" before pro-inflammatory
58. Line 347: what do the authors mean by soluble CD14
59. Line 348-349: Would benefit from rewriting.
60. Line 351: cytoplasm "in" hepatocytes "of" patients.
61. Line 352: The primary one -> One example is
62. Line 357: PAMPS also likely play "a" role in "exacerbating" inflammation in the liver....
63. Line 363: alpha-galactosylceramide in "an" acute hepatitis mouse model.
64. Line 367: Are PAMPS really structural elements of bacterial cells? It seems defining PAMPS would benefit readability.
65. Line 368: "Particularly, they produce factors" - What factors?
66. Line 368: "which main function is to damage host cells" - I think this can be rewritten as their main function is not damaging host cells.
67. Lines 372-373: "play role in worse outcomes" -> worsen outcomes
68. Line 375: "Moreover" - awkward transition
69. Line 375-377: Would benefit from rewriting.
70. Line 385-386: "led to amelioration of" -> ameliorated
71. Line 386: Remove of before labelled
72. Line 388: modulate lipid profiles how?
73. Line 390: de novo synthesis of what?
74. Line 421-422: Would benefit from rewriting.
75. Line 423: How do bile acids act on macrophages? Are these primary or secondary or both?
76. Lines 424-425: give examples
77. Line 427: give examples
78. Lines 428-431: More details would be nice
79. Line 435-436: It would be great if the authors can mention how bile acids cross the BBB. Unconjugated bile acids can pass through diffusion and unconjugated through transporters like OATPs and ASBT.
80. Line 440: them -> BAs
81. Line 449: remove "Although". "SCFAs encompass five molecules, which the shorter three by carbon length (i.e., acetate, propionate, butyrate) are dominant in the gut,...
82. Line 457: "different models" - what are these? Be specific and give examples
83. Lines 460-463: ...(1) acting locally....(2)being absorbed...(3)influencing....
84. Lines 464-472: Can combine with previous paragraph.
85. Lines 474-479: Can move to next paragraph
86. Lines 480-488: Can move to previous paragraph after line 472
87. Line 500: in animals -> in animal models
88. Line 502: some of them -> some (e.g., serotonin, melatonin)
89. Lines 502-505: Would benefit from rewriting.
90. Line 508: ...and their microbiota -> and their microbiota composition
91. Line 508: Describe tryptophan metabolic pathways that lead to the biogenesis of indoles and related products, which are the next few sections.
92. Line 514: aromatic hydrocarbon receptor -> aryl hydrocarbon receptor
93. Line 518: What is the control group?
94. Line 522-528: Would benefit from rewriting.
95. Line 530: Are the authors referring to MASLD when they say "mouse models of disease"?
96. Line 532: "high-fat diet induced mouse model" - what model are the authors referring to?
97. Line 532: IAA attenuates "a" proinflammatory
98. Line 533: Moreover, "IAA supplementation" leads to "an" AhR-dependent
99. Line 534: If Fasn was a gene that was measured, spell out and italicize
100. Line 548: Combine with previous paragraph
101. Line 551: specify what "this metabolite" is
102. Line 553: Is TMAO only produced in humans? Should rephrase
103. Line 554: "While" TMAO was first studied in the context of cardiovascular diseases (232), it has also been implicated in other conditions, such as MASLD (233,234).
104. Line 560: TMAO is suggested to bind "to the" endoplasmic reticulum ...
105. Line 563: remove various; proinflammatory states -> proinflammation
106. Line 570: Similarly to -> Similar to
107. Line 573: glucose metabolism disorders -> glucose metabolism-related disorders
108. Lines 582-583: Would benefit from rewriting.
109. Line 589: "The" amino acid L-asp is suggested to have protective roles in metabolic liver diseases including MASLD and MASH
110. Line 593: leads -> led
111. Lines 593-594: suggesting "increased absorbance in the small intestine"
112. Line 596: specify the range of direct effects on hepatocytes to indirect effects through platelets or hormones
113. Lines 599-600: Would benefit from rewriting.

114. Line 602: metabolic parameters like what?
115. Line 608: it -> PEA
116. Line 608-612: Would benefit from rewriting.
117. Line 616: Another compound that is -> Histamine is normally produced by
118. Line 617: In the gut, producers that carry the histidine decarboxylase gene such as members of ...., convert L-histidine....
119. Line 634: leads to a lowering of -> lowered
120. Line 638: it -> succinate
121. Line 639: circulating succinate concentration increase -> increased circulating cussinate concentration
122. Line 640-641: Would benefit from rewriting.
123. Line 659-660: Hence, instead of microbiota composition themselves, shifting the focus to microbial metabolites may be a more feasible therapeutic target.

Reviewer #1

This manuscript provides a review of how specific gut microbiota impacts fatty liver disease progression with a specific focus on bacterial genera/species and importantly, brief overviews of archaea, eukaryota and bacteriophages. The review also provides explanations on how microbial elements (TLR signalling molecules, bile acids and small molecules) may impact the liver in fatty liver disease. While the review is well-written, and covers a broad range of bacterial species and microbial metabolites, several issues regarding the ambiguity of the claims and the citations should be addressed.

We would like to thank the Reviewer for his assessment, and we addressed all the comments as specified below.

Major comments:

1. Lines 101-104: The role of B. stercoris is discussed. In its current form, it appears that this finding is from both references (ref) [24] and [25]. However, it is only the study in ref [25], where mouse experiments were performed and subsequent hypothesis regarding B.stercoris driving pathogenesis through LPS and BCAA. Ref [24] only mentions elevated B.stercoris in MASLD human cohort and its positive correlation to BA metabolism species. This should be rectified in the manuscript.

We are grateful to the Reviewer for pointing this out. We agree that the original wording could incorrectly suggest that both references [24] and [25] demonstrated the causative role of *B. stercoris* in MASLD pathogenesis. We have revised the text to clearly distinguish the findings of the two studies. The manuscript now states that reference [24] reports an increased abundance of *B. stercoris* in a human MASLD cohort, whereas the randomized clinical trial in reference [25] replicated this observation, demonstrated the effects of resistant starch intervention, and provided evidence from mouse experiments supporting a causative role of *B. stercoris* together with the proposed LPS- and BCAA-mediated mechanism.

2. Lines 116-117: The abundance of Prevotella copri has been associated with fibrosis in MASH in four independent cohorts. While these are indeed independent cohorts, 3 out of 4 are either primarily [29,30] or predominantly hispanic [31]. In ref [32], while not predominantly hispanic, the information about ethnicity was not corrected for as a confounding factor. Therefore there are 2 points to clarify:

a) Strain specificity: It is worthwhile to have a look at this review on P. copri here: https://doi.org/10.1080/19490976.2023.2249152, so as to understand the strain specific effects of this bacteria.

b) Ethnicity and diet composition may influence gut microbial composition as well as any bacterium's phenotype, as has been shown by numerous papers before. In the absence of references to studies that have looked beyond hispanic populations, it may not be prudent to assert the association of P. copri to fibrosis. The authors should provide references to such studies to support the assertions. If such studies do not exist, then this aspect of soft association of ethnicity to P. copri - fibrosis should ideally be acknowledged in the review.

We appreciate the Reviewer's insightful comment. We agree that the interpretation of the association between *Prevotella copri* and fibrosis requires additional context. Accordingly, we have revised the manuscript in two ways. First, we have incorporated the suggested review describing the substantial strain-level genetic and functional diversity of *P. copri*, which may explain its context-dependent effects in metabolic diseases. As deep discussion heterogeneity of *P. copri* is not within the scope of this review, we added citations to other works describing it in detail. Second, we now acknowledge that three of the four cohorts reporting an association between *P. copri* abundance and fibrosis consisted primarily of predominantly of Hispanic participants, while ethnicity was not addressed as a potential confounding factor in the remaining study. We therefore added a statement emphasizing that these findings should be interpreted with caution and that ethnicity, dietary habits, and strain-specific characteristics may all influence the observed association. We also note that studies investigating the genus *Prevotella* in MASLD have reported inconsistent findings across different populations, further supporting the need for cautious interpretation of the currently available evidence.

3. Lines 261-262: "Faecalibacterium, Subdoligranulum, Haemophilus, and Roseburia were positively correlated with MASLD". Contrasting observation, given the prior section on Faecalibacterium in the review. The authors should state here a bit more about contrasting observations. On further reading of the reference [53], this sentence appears to be misframed. These 4 genera are in fact negatively associated with MASLD. Therefore, the concerned sentence should be amended.

We thank the Reviewer for this careful observation. We agree that the original sentence was incorrectly phrased. Reference [53] reports that *Faecalibacterium*, *Subdoligranulum*, *Haemophilus*, and *Roseburia* are negatively, rather than positively, associated with MASLD. We have corrected this error and moved the statement to the section discussing hepatoprotective taxa, where it better reflects the biological context and is consistent with the previous discussion of the hepatoprotective role of *Faecalibacterium*.

4. Lines 276-291: The contrasting roles of Eubacterium, Agathabacter and Bifidobacterium have been discussed. This maybe due to co-occurrence in microbial communities. It has been shown by several studies know singular bacterial strains cannot always alone drive gut microbial homeostasis, rather it is the communities that do so. While the authors have acknowledged this fact in one sentence at the end of the section, this aspect deserves a brief overview in the context of MASLD.

We thank the Reviewer for this insightful comment. We agree that the contrasting associations reported for several bacterial taxa may reflect the importance of microbial community context and interactions between co-occurring microorganisms. We have expanded this section to include a brief discussion emphasizing that individual bacterial taxa may not independently determine disease outcomes, and that their effects can depend on microbial community structure, host factors, and environmental conditions. We have also revised the concluding statement of the section to better reflect the concept that MASLD progression is shaped by collective microbial functions rather than individual taxa alone.

5. Lines 367-371: Section on cytolysins is missing citations.

We thank the Reviewer for pointing this out and apologize for this oversight. The appropriate references have now been added to this section to support the statements regarding cytolysins.

6. Line 368: I would argue that the main function of cytolysins is not only damaging host cells, but also dissemination and competition with other microbes for niche colonization. Source: https://doi.org/10.1099/mic.0.001154

We thank the Reviewer for this valuable suggestion. We agree that cytolysins have broader biological functions beyond host cell damage, including facilitating bacterial dissemination and competition with other microorganisms for niche colonization. We have revised the text accordingly and cited the recommended reference.

Minor comments:

1. Line 87: The authors state that they "systematically discuss taxa with the most evidence", however no criteria are provided for how evidence was evaluated or how taxa were selected for inclusion. The authors should either define their selection criteria explicitly, or reframe this as a narrative synthesis reflecting the authors' expert judgement rather than implying a systematic process.

We thank the Reviewer for this important comment. We agree that the term "systematically" could imply a formal systematic review methodology, which was not the intention of this

manuscript. We have therefore revised the text to clarify that this section provides a narrative overview of selected bacterial taxa that has been most extensively investigated in relation to MASLD and its progression.

2. Couple of grammatical errors:

Line 21: "..we put particular emphasis on.."

Line 368: "Particularly, they produce factors, whose main function is to damage host cells." Lines 598-599: "L-Cit is a non-essential amino acid, an intermediate in urea cycle and also by-product of nitric oxide synthesis from L-arginine in kidneys."

We appreciate the Reviewer's careful reading of the manuscript. All suggested grammatical corrections have been implemented.

Reviewer #2

General evaluation

This review addresses a timely and important topic: the contribution of the gut microbiota and microbiota-derived metabolites to the development and progression of metabolic dysfunction-associated steatotic liver disease (MASLD). The manuscript covers a broad range of microbial components, including bacteria, archaea, fungi, bacteriophages, microbial structural products, bile acids, short-chain fatty acids, amino-acid derivatives, and other metabolites. This breadth is particularly valuable. In my opinion, the manuscript requires only minor revisions.

We thank the reviewer for the positive comments. We addressed all of them as specified in the point-by-point response.

Minor comments

• Throughout the manuscript, it is not always clear whether the reported findings derive from human cohorts, animal models, or in vitro experiments. The authors should specify the experimental context more consistently and clearly distinguish observational associations in patients from causal or mechanistic evidence obtained in experimental models.

We have revised the manuscript and added more details to descriptions to make them more specific as Reviewer suggested.

• The manuscript states that supplementation with Lactobacillus acidophilus for 14 days reduced circulating liver-enzyme activity in a small human cohort and cites reference 75. However, reference 75 is Wu et al., "Lactobacillus acidophilus ameliorates cholestatic liver

injury through inhibiting bile acid synthesis and promoting bile acid excretion," which does not describe a 14-day human MASLD intervention. Please correct this citation and verify that the other references accurately support the corresponding statements.

We thank the Reviewer for this insightful comment and for the meticulous check of our references. We agree that the original wording may have unintentionally suggested that the cited evidence referred specifically to a MASLD cohort, whereas the described intervention was conducted in a different liver disease context. To avoid this ambiguity, we have revised the text to clearly distinguish findings obtained in MASLD from those reported in other liver diseases, specifically cholestatic liver injury.

In addition, we identified an incorrect citation in the final sentence of this section, where reference 75 had been mistakenly retained. This has now been corrected and replaced with the appropriate reference. We have also carefully rechecked the references throughout this section to ensure that each citation accurately supports the corresponding statement.

• The SCFA section presents these metabolites as broadly protective, whereas their effects appear context-dependent and vary according to the individual SCFA and the biological compartment measured. For example, circulating propionate and butyrate were positively associated with significant fibrosis in a biopsy-characterized MASLD cohort cited by the authors (Thing et al., 2024). In addition, microbiota-derived acetate can support hepatic de novo lipogenesis under high-fructose conditions (Zhao et al., 2020). Please nuance this section accordingly.

Thank you for this important comment. We have substantially revised the SCFA section to provide a more balanced and nuanced discussion. Specifically, we incorporated the findings of Thing et al. (2024), showing divergent associations of circulating SCFAs with MASLD and fibrosis, including positive associations of circulating propionate and butyrate with significant fibrosis. We also incorporated the findings of Zhao et al. (2020), demonstrating that microbiota-derived acetate can serve as a substrate for hepatic de novo lipogenesis through hepatic ACSS2 under conditions of high fructose exposure. Furthermore, we added data from a human NAFLD cohort linking higher fecal acetate and propionate concentrations with an immune phenotype associated with NASH.

We also added information about SCFA synthesis. As there are many producers of SCFA and human gut and regulation is comples, we additionally refer to review that describes this in detail.

• Bacteroides is repeatedly misspelled as "Bacterioides" (lines 89, 94, and 109).

We thank the Reviewer for identifying these typographical errors. The spelling has been corrected throughout the manuscript.

• Line 329: LPS should be described more precisely as a component of the outer membrane of Gram-negative bacteria rather than generically as a bacterial "cell-wall" component.

We appreciate the Reviewer's suggestion. The description has been revised accordingly to accurately state that LPS is a component of the outer membrane of Gram-negative bacteria.

• Line 370: "b-barrel" should be replaced with "β-barrel."

We thank the Reviewer for pointing out this typographical error. The text has been corrected accordingly.

Reviewer #3

The manuscript by Wysokinska et al provides a review on the contribution of the gut environment in shaping fatty liver diseases. In particular, the authors focus on metabolic-associated steatotic liver disease (MASLD) and related diseases. The current evidence provided are mostly associative, which are limited by study design in nature. Despite the comprehensive body of work that highlights a clear association between the gut environment and MASLD, the manuscript lacks key details and would benefit from multiple rounds of formatting and cleaning up in writing style (lengthy sentences) and grammar to improve readability.

We appreciate the comments from the reviewer, and we have addressed both the writing style, and we added additional details to improve the manuscript.

Major and minor comments:
1. Line 21: typo: "we particular"

We thank the Reviewer for identifying this typographical error. The text has been corrected.

2. Line 68: It seems that gut microbiome fits better for this review than gut microbiota since gut microbiome encompasses is the collection of microorganisms and their genes, whereas microbiota limits to the living microorganisms.

While it is true that many scientists use word ‘microbiome’ broadly, and it could be used throughout our manuscript, we decided to use the word ‘microbiota’ to describe the collection of microorganisms, while the word ‘microbiome’ to when we discuss their genomic content.

3. Line 100: is this really enzyme activity or abundance?

We would like to thank the reviewer for pointing this out. We corrected this to “enzyme activity”.

4. Lines 102-104: Would benefit from rephrasing.

We appreciate the Reviewer's suggestion. This paragraph has been rephrased to improve clarity.

5. Lines 106-107: specify which "desirable features in in vitro assays"

We thank the Reviewer for pointing out this lack of specificity. The sentence has been revised to explicitly describe the beneficial properties of the *Bacteroides sp*. 4_1_36 (B6) strain observed in vitro.

6. Line 114: Is folic acid biosynthesis really upregulated or is this at the pathway level from microbial gene abundance.

We thank the Reviewer for this important comment. We have rephrased text to make it explicit that levels of folate were higher.

7. Line 118: "in some studies" - specify.

The paragraph was rephrased to clearly present sources.

8. Line 119 and onward: overuse of "interestingly" and "importantly". If something is interesting or important, specify why. I counted 14 "interestingly" and 10 "importantly". Overuse of "Moreover"

Thank you for showing the recurring pattern. We have revised the text, accordingly, replacing these adverbs with more specific wording or, where appropriate, restructuring the sentences to avoid unnecessary emphasis and improving the overall flow of the manuscript.

9. Line 124: Would benefit from describing ethanol consumption in exacerbating MASLD. The authors state the carryover of NAFLD to MASLD in terminology. NAFLD does not include alcoholic fatty liver disease or fatty liver disease from excessive alcohol consumption.

We thank the Reviewer for this comment. The discussed studies focus on moderate alcohol consumption as a factor associated with alterations in gut microbiota. Moderate alcohol consumption does not automatically mean alcohol-associated liver disease (ALD) diagnosis. Diagnostic criteria for MASLD allow alcohol consumption lower than 140 g/week for women and lower than 210 g/week for men. Our point is to show that alcohol consumption impacts microbiome composition shifting levels of *Prevotella*. And then enrichment of *Prevotella* leads to worse liver outcomes.

10. Line 131: Does R. farcis metabolize primary bile acids? In addition, specify how secondary bile acids influence inflammatory and fibrotic processes in the liver.

We thank the Reviewer for raising this point. To our best knowledge, the ability of *R. faecis* to directly metabolize primary bile acids in vivo has not been specifically demonstrated. The paper we cited to only report that intervention with *Ruminococcus faecis* leads to increase in secondary bile acids, however mechanism is unknown, hence we do not describe it.

11. Line 134-135: "improved MASLD parameters" - specify.

We appreciate the Reviewer's suggestion. We have revised the sentence to specify that the reported improvement refers to serum markers of liver injury in patients with MASLD undergoing time-restricted feeding. We have also retained the specific metabolic and liver-related parameters reported in the subsequent sentence describing the mouse model.

12. Line 146: Does HiAlc-Kpn correlate to circulating and hepatic ethanol levels?

We thank the Reviewer for this comment. We have added information regarding the association of HiAlc-*Kpn* colonization with increased circulating and hepatic ethanol levels.

13. Lines 148-151: Would benefit from rewriting. Was there increased ethanol detected in the liver?

We thank the Reviewer for this suggestion. We have revised the text to clarify that increased ethanol levels were detected in the blood of mice colonized with HiAlc-*Kpn*, rather than directly in the liver. We have also rephrased the following sentences to better distinguish bacterial ethanol production from its hepatic effects.

14. Line 155: Give examples.

We thank the Reviewer for this suggestion. We have expanded this sentence by providing examples of the observed effects of HiAlc-*Kpn* on intestinal permeability, including increased permeability markers (DAO and serum D-lactate) and reduced expression of tight junction proteins (occludin and ZO-1).

15. Line 166: The comparison is between obesity and MASH. What about non-MASLD (healthy controls) vs MASLD or healthy controls vs MASH?

We thank the Reviewer for this observation. We have added details to the manuscript to make these comparisons clearer.

16. Lines 167-169: Would benefit from rewriting.

Rewritten as suggested to improve clarity and readability.

17. Lines 173-174: Can split in 2 sentences.

The sentence has been split into two sentences as suggested to improve readability.

18. Lines 183-186: Would benefit from rewriting.

Rewritten as suggested to improve clarity and readability.

19. Lines 192-193: Would benefit from rewriting.

Rewritten as suggested to improve clarity and readability.

20. Line 209: "Amuc_1100 interacts with TLR2" - specify what the authors mean by interact. Does Amuc_1100 bind to TLR2 or indirectly regulates TLR2 through another mechanism?

'interact' means it binds to TLR2 and activates it, as it is a TLR2 agonist (proven by crystal structure https://pdbj.org/mine/summary/6lad). This is a common word to describe ligand – receptor binding (ligand-receptor interactions).

21. Line 221-223: Would benefit from rewriting.

Rewritten as suggested to improve clarity and readability.

22. Line 226: Spell out FGF2.

FGF2 was spelled out when it was first mentioned in the text.

23. Line 231: "Because they are used as probiotics" -> something like: As Lactobacillus sp are currently classified as probiotics.

Revised as suggested.

24. Line 232: "In a small human cohort" - specify how many individuals.

The information has been added.

25. Line 233: related to reference 75, in what condition are the authors referring to?

The condition has been clarified in the preceding text.

26. Line 239: "reduction of hepatocyte necrosis" -> "reduction in hepatocyte necrosis"

Revised as suggested.

27. Line 249-251: Rewrite.

The paragraph was rewritten to make it clearer.

28. Line 251: How was Helicobacter hepaticus introduced? Through oral gavage?

The information has been added.

29. Line 252-255: Would benefit from rewriting.

The paragraph was rewritten to make it clearer.

30. Line 255-257: Would benefit from rewriting.

The paragraph was rewritten to make it clearer.

31. Line 256: What is the sample size?

The information has been added.

32. Line 261: In the previous section, the authors state Faecalibacterium is negatively correlated with MASLD.

We have corrected this inconsistency. The previous statement contained a typographical error; the intended meaning was that *Faecalibacterium* is positively correlated with MASLD.

33. Line 264 and in all sections: via does not need to be italicized.

Thank you for pointing this out. We initially italicized via, similarly, to other Latin terms such as in vitro and de novo. However, considering the widespread use of "via" in English scientific writing, we have followed the reviewer's suggestion and removed the italics throughout the manuscript.

34. Line 264: ...via their proinflammatory potential -> something like via their contribution in creating proinflammatory environment. Give examples.

Thank you for this valuable comment. We revised this section to state that Streptococcus (including *S. salivarius* and *S. parasanguinis*), representatives of *Enterobacteriaceae*, and *Megasphaera* are enriched in MASH patients and have been proposed as microbial markers of disease severity. We also clarified that underlying mechanisms and causal relationships remain to be elucidated.

35. Line 279: However, in the cohort -> However in a cohort.

We respectfully disagree with this suggestion and have retained the original wording, "However, in the cohort," as we believe it is grammatically appropriate.

36. Line 279: What is the sample size?

Throughout the whole manuscript, we do not specify all the details of experiments we refer to. We believe that readers who are interested in such details can check them in references.

37. Line 284: What do the authors mean by depleted? Go to 0 or decreased?

We thank the Reviewer for this comment. We agree that the term "depleted" may be ambiguous. We have revised the text to clarify that it refers to a reduction in abundance rather than complete absence.

38. Lines 286-289: Would benefit from rewriting.

The passage has been rephrased to improve the clarity and structure of the text.

39. Line 292: make consistent headers. The first header was bacteria associated with MASLD with specific examples of bacteria. Archea and others should have similar headers.

The section headers have been revised to ensure consistent formatting throughout.

40. Line 300-301: Rewrite.

The flow of the paragraph was improved.

41. Line 302: lowercase "Increased"

Corrected as suggested.

42. Line 303: Are the authors referring to MASLD for "the disease"? It seems like there is overuse of pronouns (e.g., it) and unclear reference to the exact context.

We changed it to “this condition” which directs the reader to the “MASLD” from the previous sentence while not overusing the same word in text.

43. Line 304-306: Would benefit from rewriting.

The passage has been rewritten to improve clarity and readability.

44. Line 305: Spell out ALD

ALD has been spelled out when it was first mentioned and is also in the abbreviation list.

45. Line 308: "in the livers in mice" -> "mouse livers"

Revised as suggested.

46. Line 308-309: why is this important?

We have rephrased the sentence

47. Lines 314-315: Would benefit from rewriting.

Rewritten to enhance clarity.

48. Line 322: Microbial elements -> microbiota-associated metabolites

Replaced according to the suggestion.

49. Lines 323-325: Should highlight this in the introduction. The first half heavily focuses on microbial abundance associated with MASLD and the latter half focuses on functional outputs of gut microbes. The manuscript would benefit from clear distinction of these in the introduction/abstract.

We thank the Reviewer for this valuable suggestion. Although the distinction between compositional alterations of the gut microbiota and their functional consequences was

already introduced in the Abstract and Introduction, we agree that it could be stated more explicitly. Therefore, we have revised both sections to better emphasize that this review addresses two complementary aspects of gut microbiota involvement in MASLD: (i) changes in microbial composition and (ii) functional outputs, particularly microbiota-derived metabolites and their contribution to gut–liver axis dysfunction.

50. The second section suffers from the lack of key information involving microbial pathways. What microbes are involved in the metabolic pathways of metabolite production and how are their abundances altered MASLD?

We thank the Reviewer for this insightful comment. We agree that linking microbial metabolites to microorganisms and metabolic pathways responsible for their production would strengthen the mechanistic perspective. However, current evidence does not yet allow a comprehensive mapping of individual metabolites to specific microbial taxa in MASLD. Most microbial metabolites are produced by phylogenetically diverse microorganisms with considerable functional redundancy, and metabolite production depends primarily on the presence and activity of specific metabolic pathways rather than the abundance of individual taxa. To clarify this point, where supported by literature, we have included examples of microbial groups known to contribute to the production of specific metabolites.

51. Line 238: Spell out LPS

We adopted a convention to explain the full term for the first time. LPS was spelled out at its first mention (Line 104).

52. Line 333: "Microbiota of MASLD patients contain bacteria that are high-endotoxic producers" -> "Microbiota of MASLD patients showed increased abundance of endotoxic producing bacteria"

Replaced according to the suggestion.

53. Line 334-335: How was causality determined?

We added more details about the experimental setup explanation.

54. Line 341: When "the" intestinal barrier is disrupted,

Revised as suggested.

55. Line 343: "Once inside the liver LPS binds to TLR4" -> Upon translocation to the liver, LPS binds to TLR4

Actually, LPS binds to CD14, which then binds to TLR4. We rephrased the sentence to make it clearer.

56. Line 343: what do the authors mean by "adaptor"

CD14 is often described as an adaptor protein for TLR4. However, to simplify the sentence, we removed this.

57. Line 345: remove "of" before pro-inflammatory

Revised as suggested.

58. Line 347: what do the authors mean by soluble CD14

The context was added to explain the meaning of “soluble CD14”.CD14 has two forms, soluble and membrane bound. Here, the soluble CD14 performs the function of LPS binding. We now add a reference to the description of molecular biology behind LPS sensing, for those who are not familiar with it.

59. Line 348-349: Would benefit from rewriting.

Rewritten to enhance clarity.

60. Line 351: cytoplasm "in" hepatocytes "of" patients.

Revised as suggested.

61. Line 352: The primary one -> One example is

Replaced according to the suggestion.

62. Line 357: PAMPS also likely play "a" role in "exacerbating" inflammation in the liver....

Revised as suggested.

63. Line 363: alpha-galactosylceramide in "an" acute hepatitis mouse model.

Revised as suggested.

64. Line 367: Are PAMPS really structural elements of bacterial cells? It seems defining PAMPS would benefit readability.

We thank the Reviewer for this suggestion. We agree that defining PAMPs improves readability. Therefore, we revised the sentence to define PAMPs as highly conserved microbial molecules recognized by host pattern recognition receptors (PRRs), rather than describing them primarily as structural components of bacterial cells.

65. Line 368: "Particularly, they produce factors" - What factors?

We thank the Reviewer for this comment. We agree that the previous wording was insufficiently specific. The term “factors” refers to secreted virulence factors, particularly cytolysins discussed in the following sentences. We have revised the text to clarify this point and improve the flow of the paragraph.

66. Line 368: "which main function is to damage host cells" - I think this can be rewritten as their main function is not damaging host cells.

We thank the Reviewer for this comment. We agree that describing host cell damage as the main function of cytolysins was an oversimplification. We have revised the text to clarify that cytolysins have diverse biological functions, including bacterial competition, niche colonization, and host cell damage.

67. Lines 372-373: "play role in worse outcomes" -> worsen outcomes

The text has been revised for clarity and flow.

68. Line 375: "Moreover" - awkward transition

Rephrased as suggested.

69. Line 375-377: Would benefit from rewriting.

The text has been revised for clarity and flow.

70. Line 385-386: "led to amelioration of" -> ameliorated

Revised as suggested.

71. Line 386: Remove of before labelled

Revised as suggested.

72. Line 388: modulate lipid profiles how?

We thank the Reviewer for this comment. We agree that the previous statement was too general. We have revised the text to specify that B. theta-derived sphingolipids can influence hepatic lipid metabolism by modulating host sphingolipid pools and pathways related to fatty acid β-oxidation.

73. Line 390: de novo synthesis of what?

We thank the Reviewer for this comment. We agree that the previous wording was insufficiently specific. We have clarified that the statement refers to host de novo sphingolipid synthesis.

74. Line 421-422: Would benefit from rewriting.

The sentence has been rewritten to improve clarity and readability.

75. Line 423: How do bile acids act on macrophages? Are these primary or secondary or both?

Thank you for this comment. In this point we want to show that responses of cells to bile acids may be very different. Explaining interactions of bile acids and immune cells in depth is beyond the scope of this manuscript. We have expanded this section to provide specific examples of how bile acids modulate macrophage responses. We now describe the TGR5–cAMP–NF-κB pathway and its effects on pro-inflammatory cytokine production in macrophages and Kupffer cells. Moreover, we added citations to reviews that describe the immune functions of BAs in detail.

76. Lines 424-425: give examples

We have revised this section to include specific examples illustrating the distinct effects of individual bile acids on macrophage populations. The revised text now highlights differences in macrophage responses mediated by bile acids through TGR5 and NF-κB signaling.

77. Line 427: give examples

A specific example has been added showing that the secondary bile acid isoalloLCA promotes regulatory T-cell differentiation through an NR4A1-dependent mechanism.

78. Lines 428-431: More details would be nice

Thank you for this suggestion. We expanded this section to provide specific examples of how bile acids modulate intestinal barrier integrity. We added the example of cholic acid, a primary bile acid that can impair intestinal epithelial repair by suppressing intestinal stem cell renewal, as well as the example of taurodeoxycholic acid (TDCA), a secondary bile acid that can support intestinal barrier function by enhancing tight-junction protein expression through Nrf2 signaling. We also clarified the role of bile acid availability in shaping gut microbiota composition and its inflammatory potential.

79. Line 435-436: It would be great if the authors can mention how bile acids cross the BBB. Unconjugated bile acids can pass through diffusion and unconjugated through transporters like OATPs and ASBT.

We thank Reviewer for suggestion; however, we believe that describing mechanisms of BAs action outside the gastrointestinal system is outside the scope of this review.

80. Line 440: them -> BAs

Revised as suggested.

81. Line 449: remove "Although". "SCFAs encompass five molecules, which the shorter three by carbon length (i.e., acetate, propionate, butyrate) are dominant in the gut,...

Revised as suggested.

82. Line 457: "different models" - what are these? Be specific and give examples

Thank you for this helpful comment. We have revised the sentence to specify the experimental models instead of using the general term "different models." The text now states that butyrate supplementation showed beneficial effects in a Western diet-induced MASH model and an ovariectomy/high-fat diet-induced model of estrogen deficiency-associated MASLD, thereby providing specific examples of the experimental settings.

83. Lines 460-463: ...(1) acting locally....(2)being absorbed...(3)influencing....

Revised as suggested.

84. Lines 464-472: Can combine with previous paragraph.

The SCFA section has been substantially reorganized, and the previously separate paragraphs have been integrated into a new structure. The relevant content is now presented together with the newly added evidence addressing the context-dependent effects of SCFAs.

85. Lines 474-479: Can move to next paragraph

Following the broader reorganization of the SCFA section, the relevant content has been repositioned within the revised paragraph structure and integrated with the newly added evidence on the context-dependent effects of SCFAs.

86. Lines 480-488: Can move to previous paragraph after line 472

The section has been substantially reorganized, so the original paragraph boundaries no longer apply. The relevant content has been integrated into the revised SCFA section together with the newly added evidence on both beneficial and potentially detrimental/context-dependent effects.

87. Line 500: in animals -> in animal models

Revised as suggested.

88. Line 502: some of them -> some (e.g., serotonin, melatonin)

Revised as suggested.

89. Lines 502-505: Would benefit from rewriting.

Thank you for this suggestion. We have rewritten the passage to improve its clarity and readability and to better distinguish between host-derived and microbiota-derived tryptophan metabolites.

90. Line 508: ...and their microbiota -> and their microbiota composition

Revised as suggested.

91. Line 508: Describe tryptophan metabolic pathways that lead to the biogenesis of indoles and related products, which are the next few sections.

Thank you for this comment. We have added a brief description of the main microbial tryptophan metabolic pathways leading to the formation of indole and related metabolites, including the direct conversion of tryptophan to indole by bacterial tryptophanase and the distinct pathways leading to other indole derivatives. This description has been added before the sections discussing individual indole metabolites.

92. Line 514: aromatic hydrocarbon receptor -> aryl hydrocarbon receptor

Revised as suggested.

93. Line 518: What is the control group?

The information has been added.

94. Line 522-528: Would benefit from rewriting.

We appreciate the reviewer’s comment and have revised the indicated passage accordingly.

95. Line 530: Are the authors referring to MASLD when they say "mouse models of disease"?

We appreciate the reviewer’s comment. Yes, we were referring to mouse models of MASLD. We have revised the sentence to make this explicit.

96. Line 532: "high-fat diet induced mouse model" - what model are the authors referring to?

We thank the reviewer for highlighting this point. We were referring to the Western diet-based DIAMOND model of diet-induced MASLD used in reference 219. We have revised the sentence accordingly to specify the model.

97. Line 532: IAA attenuates "a" proinflammatory

Revised as suggested.

98. Line 533: Moreover, "IAA supplementation" leads to "an" AhR-dependent

Revised as suggested.

99. Line 534: If Fasn was a gene that was measured, spell out and italicize

We thank the reviewer for highlighting this point. *Fasn* was measured at the gene expression level, and we have therefore spelled out the gene name and italicized the gene symbol in the revised manuscript.

100. Line 548: Combine with previous paragraph

We thank the reviewer for this suggestion. The paragraph has been combined with the preceding paragraph as recommended.

101. Line 551: specify what "this metabolite" is

Rewritten to enhance clarity.

102. Line 553: Is TMAO only produced in humans? Should rephrase

We thank the reviewer for pointing this out. We have rephrased the sentence to clarify that TMAO is produced by the host from the bacteria-derived precursor TMA, rather than implying that TMAO is produced exclusively by humans.

103. Line 554: "While" TMAO was first studied in the context of cardiovascular diseases (232), it has also been implicated in other conditions, such as MASLD (233,234).

Revised as suggested.

104. Line 560: TMAO is suggested to bind "to the" endoplasmic reticulum ...

Revised as suggested.

105. Line 563: remove various; proinflammatory states -> proinflammation

Revised as suggested.

106. Line 570: Similarly to -> Similar to

Revised as suggested.

107. Line 573: glucose metabolism disorders -> glucose metabolism-related disorders

Revised as suggested.

108. Lines 582-583: Would benefit from rewriting.

We have rewritten the indicated passage to improve its clarity and precision.

109. Line 589: "The" amino acid L-asp is suggested to have protective roles in metabolic liver diseases including MASLD and MASH

Revised as suggested.

110. Line 593: leads -> led

Revised as suggested.

111. Lines 593-594: suggesting "increased absorbance in the small intestine"

Replaced according to the suggestion.

112. Line 596: specify the range of direct effects on hepatocytes to indirect effects through platelets or hormones

In response to the reviewer's comment, we have expanded this section to provide a more detailed description of the proposed mechanisms underlying the beneficial effects of L-Asp.

113. Lines 599-600: Would benefit from rewriting.

Rewritten to enhance clarity.

114. Line 602: metabolic parameters like what?

The reviewer's point has been addressed by specifying the metabolic parameters affected by L-Cit supplementation.

115. Line 608: it -> PEA

Revised as suggested.

116. Line 608-612: Would benefit from rewriting.

We have rewritten lines 608–612 to improve the clarity and logical flow of the paragraph

117. Line 616: Another compound that is -> Histamine is normally produced by

Revised as suggested.

118. Line 617: In the gut, producers that carry the histidine decarboxylase gene such as members of …., convert L-histidine….

Revised as suggested.

119. Line 634: leads to a lowering of -> lowered

Revised as suggested.

120. Line 638: it -> succinate

Revised as suggested.

121. Line 639: circulating succinate concentration increase -> increased circulating cussinate concentration

Revised as suggested.

122. Line 640-641: Would benefit from rewriting.

Rewritten to enhance clarity.

123. Line 659-660: Hence, instead of microbiota composition themselves, shifting the focus to microbial metabolites may be a more feasible therapeutic target.

Replaced according to the suggestion.

September 23, 2026

RE: Life Science Alliance Manuscript #LSA-2026-03756-TRR

Dr. Aleksandra A Kolodziejczyk
International Institute of Molecular and Cell Biology
Laboratory of Cellular Genomics
Ksiecia Trojdena 4
Warsaw 02-109
Poland

Dear Dr. Kolodziejczyk,

Thank you for submitting your revised Review manuscript entitled "Gut microbiota shaping fatty liver disease progression". As you will see, the reviewers are now satisfied with no further requests. We would be happy to publish your paper in Life Science Alliance pending any final revisions to meet our formatting requirements outlined below.

Please upload the following materials to our submission system by logging in to your account: https://lsa.msubmit.net/cgi-bin/main.plex
You will be guided to complete the submission of your revised manuscript.

Please add the X and Bluesky handles of your host institute/organization, as well as your own, and/or one of the authors, in our system.

These items are required for acceptance.

-- An editable version of the final text (.DOC or .DOCX) is needed for copyediting (no PDFs).

-- High-resolution figure, supplementary figure and video files uploaded as individual files: See our detailed guidelines for preparing your production-ready images, https://life-science-alliance.org/authorguide

-- Summary blurb (enter in submission system): A short text summarizing in a single sentence the study (max. 200 characters including spaces). This text is used in conjunction with the titles of papers, hence should be informative and complementary to the title. It should describe the context and significance of the findings for a general readership; it should be written in the present tense and refer to the work in the third person. Author names should not be mentioned.

The license to publish form must be signed before your manuscript can be sent to production. A link to the license to publish form will be sent to the corresponding author only. Please take a moment to check your funder requirements.

Thank you for your attention to these final processing requirements. Please revise and format the manuscript and upload materials within 7 days.

Thank you for this interesting contribution, we look forward to publishing your Commissioned Review in Life Science Alliance.

Sincerely,

Tim Fessenden
Scientific Editor
Life Science Alliance

Reviewer #1 (Comments to the Authors (Required)):

The authors have sufficiently addressed all points mentioned in my first review. I have no further comments.

Reviewer #2 (Comments to the Authors (Required)):

The authors addressed all the points that needed attention.

September 29, 2026

RE: Life Science Alliance Manuscript #LSA-2026-03756-TRRR

Dr. Aleksandra A Kolodziejczyk
International Institute of Molecular and Cell Biology
Laboratory of Cellular Genomics
Ksiecia Trojdena 4
Warsaw 02-109
Poland

Dear Dr. Kolodziejczyk,

Thank you for submitting your Commissioned Review entitled "Gut microbiota shaping fatty liver disease progression". It is a pleasure to let you know that your manuscript is now accepted for publication in Life Science Alliance. Congratulations on this interesting work.

The final published version of your manuscript will be deposited by us to PubMed Central upon publication.

Your manuscript will now progress through copyediting and proofing. Your article will publish open access upon publication under a CC-BY license.

Reviews, decision letters, and point-by-point responses associated with peer-review at Life Science Alliance will be published, alongside the manuscript. If you do want to opt out of having the reviewer reports and your point-by-point responses displayed, please let us know immediately.

IMPORTANT: If you will be unreachable at any time, please provide us with the email address of an alternate author. Failure to respond to routine queries may lead to unavoidable delays in publication.

Scheduling details will be available from our production department. You will receive proofs shortly before the publication date. Only essential corrections can be made at the proof stage so if there are any minor final changes you wish to make to the manuscript, please let the journal office know now.

DISTRIBUTION OF MATERIALS:
Authors are required to distribute freely any materials used in experiments published in Life Science Alliance. Authors are encouraged to deposit materials used in their studies to the appropriate repositories for distribution to researchers.

You can contact the journal office with any questions, contact@life-science-alliance.org

Again, congratulations on a very nice paper. I hope you found the review process to be constructive and are pleased with how the manuscript was handled editorially. We look forward to future exciting submissions from your lab.

Sincerely,

Tim Fessenden
Scientific Editor
Life Science Alliance
